# Understanding food loss patterns across developed and developing countries using a GDP, growth rate, and health expenditure-based typology

**DOI:** 10.1038/s41598-025-13156-3

**Published:** 2025-07-29

**Authors:** Buse Baykoca, Salim Yılmaz

**Affiliations:** 1 Department of Nutrition and Dietetics, Graduate School, Istanbul Okan University, Istanbul, Türkiye; 2 Department of Health Management, Faculty of Health Sciences, Acibadem Mehmet Ali Aydinlar University, Istanbul, 34752 Türkiye

**Keywords:** Food loss, Sustainable food systems, Development classification, Socioeconomic indicators, Multilevel modelling, Environmental social sciences, Sustainability, Health policy

## Abstract

Food loss and waste (FLW) threaten progress toward Sustainable Development Goals (SDG) 12.3, yet their distribution by development stage remains under-quantified. We created a time-weighted K-means typology for 105 countries (2000–2022) using Gross Domestic Product (GDP) per capita, GDP growth, and per-capita health expenditure—indicators chosen to capture economic capacity, growth momentum, and institutional investment. The scheme classified nations as developed (*n* = 13), developing (*n* = 92), or hybrid, with > 98% membership stability across weighting parameters. Linking this typology with FAO’s FLW data, we modelled food loss percentages (FLP) across ten commodity groups and eight supply-chain stages using multilevel mixed-effects regression. Developed countries lost the most food at consumption (22.5%), dwarfing developing (6.8%) and hybrid cases (9.0–14.2%), whereas developing nations suffered greater upstream losses at harvest/on-farm (3.7%). FLP in developing economies was significantly lower for grains (β = − 8.02, *p* = 0.007), oilseeds (β = − 19.29, *p* = 0.016) and pulses (β = − 5.43, *p* = 0.021). From 2000 to 2022, oilseed and sugar losses rose (β = 0.26, *p* < 0.001), while roots/tubers and dairy/eggs declined (β = − 0.31, − 0.89; *p* < 0.01). Stage analyses revealed pronounced development gaps at consumption (β = − 16.06, *p* < 0.001) and processing (β = − 5.58, *p* = 0.014), alongside a rising trend in marketing/retail losses (β = 0.25, *p* = 0.005). Country-level random effects explained up to 90% of variance, underscoring the dominance of local conditions. The evidence supports consumer-behaviour interventions in high-income settings, upstream infrastructure investment in developing regions, and dual-track strategies in hybrids. Our typology provides a scalable, policy-ready lens for designing targeted FLW actions aligned with SDG 12.3.

**Supplementary Information:**

The online version contains supplementary material available at 10.1038/s41598-025-13156-3.

## Introduction

The United Nation’s (UN) 2015 Sustainable Development Goals’ (SDG) include Target 12.3—halving food waste and cutting supply-chain losses by 2030—making Food Loss and Waste (FLW) reduction critical for sustainability, food security, and resource efficiency worldwide^1^. Globally, approximately 1.3 billion tons of food are lost or wasted each year—an amount equivalent to one-third of total food production. FLW not only leads to substantial economic losses but also contributes to approximately 8% of global greenhouse gas emissions, resulting in serious environmental harm^2^. Here, “food loss” refers to reductions in edible food mass during production, post-harvest handling, and processing stages, while “food waste” occurs at the retail and consumer levels, mostly due to behavioral factors^3^. In 2021, 13.2% of global food production was lost during post-harvest stages of transport, storage, wholesale, and processing, while in 2022, 19% of all food produced was wasted at the retail and consumer levels^3,4^. Altogether, 1.05 billion tons of edible food were wasted, approximately 60% of which originated from households^5^. This situation reveals a stark contradiction: roughly 1 billion edible meals are discarded daily while 783 million people worldwide suffer from hunger^2,4^. FLW exacerbates food insecurity by limiting physical and economic access to food, particularly in vulnerable regions^4^.

FLW is concentrated at different stages of the supply chain in developed and developing countries. According to the Food and Agriculture Organization (FAO) report^3^losses in developed countries occur predominantly at the retail and consumer levels; in contrast, in developing countries, they are more concentrated in the earlier stages of production, storage, transportation, and processing. Furthermore, while approximately 30% of total food production is wasted in developed countries, this proportion can reach up to 40% in developing nations. These figures indicate that FLW varies both in magnitude and structure across different stages of the supply chain^2,3^. However, despite these structural differences, much of the literature approaches food waste from a narrow geographical or commodity-based perspective^6^. Most studies focus on a single country or region, lacking comparative global-level analyses^7,8^. For instance, the findings of a study analyzing how food waste in Kampala is distributed across different institutional structures are limited to the regional context^7^. Similarly, some research examines a single commodity, such as poultry, to determine the causes of waste through consumer behavior^9^. These geographically or product-specific studies fail to capture the broader structural dynamics of food waste on a global scale, limiting the ability to formulate holistic policy interventions.

Hybrid-type countries—those exhibiting economic and infrastructural characteristics at the intersection of developed and developing economies—present a unique and critical context for understanding FLW dynamics. These countries often possess advanced supply-chain infrastructures within urban areas, yet simultaneously grapple with persistent inefficiencies typical of rural or transitional regions, leading to significant FLW at multiple stages of the food supply chain. For instance, in Republic of Korea, approximately 48% of FLW arises during upstream stages (production and processing), while the remaining 52% occurs downstream at the retail and consumer level^10^. In New Zealand, retail sectors alone contribute substantially to food waste, estimated at approximately 13 kg per capita annually, with significant waste occurring in fresh produce, bakery items, and meat products due to strict quality standards, logistical inefficiencies, and inadequate waste diversion infrastructure^11^. These hybrid dynamics underscore complex interactions between economic indicators such as Gross Domestic Product (GDP) per capita, economic growth rates, and health expenditures, all of which directly and indirectly influence FLW patterns. Rapid economic growth without parallel developments in infrastructure and institutional capacity often exacerbates post-harvest losses due to logistical bottlenecks and inadequate storage facilities^12^. Additionally, rising per capita health expenditure, indicative of enhanced institutional capacities and higher consumer standards for food safety, may paradoxically contribute to higher FLW at retail and consumer levels due to stringent quality standards and increased food discard rates^11,13–15^. Furthermore, the environmental repercussions of such waste are profound, involving substantial CO₂ emissions, water footprints, and energy usage. In New Zealand alone, food waste contributes approximately 4.2 million tonnes CO₂-equivalent emissions and consumes 4.7 billion cubic meters of water annually^11,16^. These environmental impacts highlight the urgent need for multi-scalar and systemic interventions that address FLW as more than just a technical inefficiency but as a socio-economic and institutional challenge requiring comprehensive policy responses^17^. Therefore, examining global FLW patterns through a multidimensional classification based on GDP per capita, GDP growth rate, and health expenditure per capita, rather than traditional income-based classifications, enables a more nuanced understanding of the underlying socio-economic and institutional factors influencing food-loss dynamics^2,14,17^. Such an approach provides granular insights critical for designing targeted policy interventions aimed at reducing food loss, enhancing supply chain efficiency, and promoting sustainable development in both transitional and established economies.

This study aims to investigate global food-loss patterns through a novel multidimensional classification of countries based on their development status, utilizing GDP per capita, GDP growth rate, and health expenditure per capita. Unlike conventional income-based classifications by organizations such as the World Bank or United Nations (UN), this approach explicitly captures the dynamic and structural factors underpinning socioeconomic development and institutional capacity, which critically influence food-loss outcomes. Additionally, recognizing that historical economic conditions exert lasting impacts on current food-loss patterns, we introduce an exponential temporal weighting mechanism to optimally balance recent economic developments against historical trends. Through rigorous sensitivity analyses, we confirm the stability and robustness of our classification, identifying borderline cases *(New Zealand and the Republic of Korea)* whose classification sensitivity highlights transitional or hybrid economic characteristics driven by recent versus historical economic performance. By combining this empirically validated development classification with detailed commodity- and supply-chain-specific food-loss data from FAO, the study provides granular insights into the intersections between national development trajectories and food-loss dynamics, ultimately guiding targeted policy interventions.

## Methods

This study, as an empirical secondary data analysis, investigates global food loss and waste patterns within a multilevel and multivariate analytical framework. To construct an empirically grounded typology of countries by development level, we employed an unsupervised K-means clustering algorithm using three core socioeconomic indicators: GDP per capita, GDP growth rate, and health expenditure per capita^18–21^. Food loss is closely linked to economic resources, production efficiency, the level of agricultural modernization, infrastructure quality, and the effectiveness of food security systems^17,22–24^. GDP per capita reflects the fundamental economic capacity and investment potential that determine a country’s ability to adopt modern equipment and technologies in agricultural and food production processes^22–24^. GDP growth rate captures recent economic momentum and structural transformations, signaling the scale of recent agricultural modernization efforts and infrastructure investments^25,26^. Health expenditure per capita serves as a proxy for institutional capacity, representing investments in strengthening food safety standards and health systems, both of which are critical for maintaining supply chain integrity and minimizing losses^14,17,22^.

Given the cumulative effects of these indicators over time, we integrated an exponential temporal weighting function into our analysis. This approach preserves the long-term influence of historical economic conditions while placing greater emphasis on more recent years. A comprehensive sensitivity analysis of the weighting parameter (λ = 0.00–0.50) identified λ = 0.20 as the optimal value, yielding a stable and methodologically robust two-cluster solution that classified countries as “developed” (*n* = 13) or “developing” (*n* = 92). This classification was further validated by identifying borderline countries—New Zealand (close to the developed cluster with early-period data) and the Republic of Korea (near the developing cluster with predominantly recent data). These two cases, with their limited temporal coverage and differing trajectories of economic transformation, were characterized as transitional or hybrid economies. Their profiles provide supporting evidence for theoretical assumptions about how historical economic conditions shape contemporary food loss patterns.

By integrating this empirically validated development-level classification with FAO food loss percentage (FLP) data, we analyzed patterns of food loss across commodity groups and supply chain stages using multilevel mixed-effects models. The inclusion of the covariate (Year – 2000) controlled for historical trends in food loss, reducing potential biases associated with the irregular and fragmented temporal coverage identified during the missing data analysis. This methodological framework provides a robust basis for examining how diverse economic development trajectories and temporal dynamics shape global food loss patterns.

A unified dataset was constructed by integrating countries’ economic and health indicators with food loss data. GDP per capita in United States Dollars (USD), GDP growth rate, and per capita health expenditure were obtained from the World Bank, while food loss percentage data were sourced from FAO’s FLW database. Differences in country naming conventions between the two sources were carefully examined, and names were manually cross-checked and harmonized to ensure consistency. This matching process identified 105 countries common to both datasets, providing a sufficiently robust and representative sample for analysis. The relevant variables were consolidated into a unified dataset containing economic and health indicators alongside FLP values for comprehensive modeling^27^.

Prior to model estimation, a systematic assessment of missing data was conducted to evaluate the extent, distribution, and potential implications of missingness in the dataset. The initial dataset comprised 25,776 observations; however, after accounting for missing country, commodity, and activity information, the analyzable sample size was reduced to 24,314 observations. Missing values were summarized across key variables (commodity, activity, and FLP), years, and countries. Approximately 14.4% of commodity and FLP observations and 24% of activity labels were missing, with gaps concentrated in earlier years and in countries with limited reporting capacity. To ensure robust modeling and avoid biased estimates due to sparse data, commodities with insufficient temporal and geographic coverage were excluded from the analysis. Notably, beverages were removed because of pervasive missingness across countries and years, resulting in an effective reduction of the commodity groups analyzed from 11 to 10. A similar screening was applied to activity groups, but all eight categories retained sufficient coverage for modeling. Year-wise coverage, country-level data availability, and observation counts per commodity–activity combination were also examined to assess temporal and geographic representativeness. Overall, 85.6% of the total dataset retained complete FLP information, and listwise deletion was applied during model fitting. No imputation was performed because the missingness patterns reflected structural reporting gaps rather than random data loss. Supplementary Material (*Tables S1–S4 and Figures **S1**–S3*) provides a detailed account of these patterns and their implications for model robustness.

The integrated dataset compiled for this study spans multiple years, introducing substantial temporal heterogeneity and raising the potential for disproportionate influence from earlier years and countries with denser historical records—particularly developed economies with longer data series. To address these imbalances during the construction of the development level typology, we applied a custom exponential weighting function exclusively within the clustering procedure. This weighting approach allowed the classification algorithm to give greater emphasis to recent values of GDP per capita, GDP growth rate, and health expenditure per capita—key indicators reflecting economic resources, recent economic momentum, and institutional capacity—while still accounting for the cumulative legacy effects of past economic trajectories.

$$\:{w}_{t}=\frac{{e}^{\lambda\:*\left[{t}_{i}-\text{min}\left(\text{t}\right)\right]}}{{\sum\:}_{s\in\:t}{e}^{\lambda\:*\left[{s}_{t}-\text{min}\left(\text{t}\right)\right]}},\:\lambda\:>0$$

where;

- **e**: Euler’s number;
- **t**_**i**_: the year of the corresponding observation;
- **min(t)**: the minimum year within the available observations used to normalize the earliest time point to zero;
- **λ**: a positive parameter (λ > 0) that controls the slope of the weighting function over time;
- **s**_**t**_: summation, which is performed over all available years (index *s*).

The λ parameter in the weighting function was selected through a sensitivity analysis of silhouette widths across λ values ranging from 0.00 to 0.50. This analysis identified λ = 0.2 as the configuration yielding the highest mean silhouette width (0.699), indicating the most consistent and well-separated clustering structure. At λ = 0.2, the optimal number of clusters was determined to be k = 2, which aligns with the conceptual aim of distinguishing “developed” and “developing” countries. Although models with λ ≥ 0.25 began suggesting a three-cluster solution, this alternative was not retained. Preliminary missing data diagnostics revealed insufficient data coverage and internal consistency within the additional subgroup, raising concerns about its empirical validity. Moreover, the subgroup lacked clear socioeconomic distinctiveness, and its small size limited statistical power and interpretability in subsequent modeling. To strengthen confidence in the two-cluster solution observed at λ = 0.2, we performed an additional validation using the elbow method to evaluate within-cluster sum of squares (WCSS). This secondary assessment confirmed k = 2 as the inflection point beyond which further increases in k yielded diminishing returns. Together, the silhouette and elbow analyses provide complementary evidence supporting the selection of λ = 0.2 and k = 2 as the most robust methodological configuration. This configuration reduces the overrepresentation of historical data from developed countries (particularly older records of GDP per capita, GDP growth rate, and health expenditure per capita) and more accurately reflects contemporary development levels. After fixing λ = 0.2 as a hyperparameter within the optimized weighting scheme, the clustering procedure delineated two empirically stable groups—“developed” (*n* = 13) and “developing” (*n* = 92)—which form the analytical foundation for subsequent modeling of food loss patterns.

Importantly, this temporal weighting was confined to the clustering phase. In subsequent modeling of food loss percentages, no weighting of years was applied; instead, temporal effects were controlled using a linear covariate (Year – 2000) within the multilevel mixed-effects framework. This decision was empirically supported by a sensitivity analysis (*Supplementary Material*,* Chap. 2*), where we compared time-weighted cross-sectional models with models including (Year – 2000) as a covariate across commodity and activity groups using Akaike Information Criterion (AIC). The analysis consistently identified λ = 0 (no temporal weighting) as the optimal specification, reinforcing the appropriateness of modeling year effects directly within the multilevel framework. This approach also facilitated a more interpretable assessment of long-term trends in food loss, while accounting for the uneven temporal distribution of the data.

After classifying countries as either “developed” or “developing,” we incorporated year as a linear covariate and estimated separate Generalized Linear Mixed Models (GLMMs) for each of the 10 commodity groups and 8 supply chain stages, using FLP as the dependent variable. FLP was selected as the dependent variable for some reasons. First, expressing losses as a percentage of total available food, rather than as absolute tonnage, enables cross-country comparability by normalizing for differences in country size and production volumes; this allows small- and large-scale food systems to be analyzed on a common scale. Second, FLP aligns with international benchmarks, mirroring the SDG 12.3 “Food Loss Index” adopted by the FAO and United Nations (UN), which makes our findings directly interpretable within global targets and enhances their relevance for policy translation^1,4,5,19,28^. Third, FAO provides percentage-based loss data with greater coverage across commodities, years, and countries, whereas absolute-tonnage figures are often incomplete or extrapolated. Using FLP therefore ensures data consistency, minimizes list-wise deletion, and preserves statistical power in the multilevel models.

The commodity groups analyzed include grains, roots and tubers, vegetables, fruits, pulses, meat products, dairy and eggs, oilseeds, sugar products, and others^2,19^. Here, *“others”* refers to the residual category defined by FAO^19^which aggregates miscellaneous agricultural products such as spices, condiments, and minor commodities not included in the primary groups^2^. Similarly, the supply chain stages comprise harvest and on-farm operations, processing, transport and distribution, storage, marketing and retail, consumption, packaging, and others. In this context, *“others”* reflects the FAO classification of unclassified or aggregated loss activities that do not fit neatly into the main stages^2,19^. The model structure, which accounts for countries (c), commodity group (g) or supply chain stage (s), development status (D), and time (t), is summarized by the following equation:

$$\:{FLP}_{c,g\left(s\right),t}={\beta\:}_{0g\left(s\right)}+\:{\beta\:}_{1g\left(s\right)}{D}_{c}+{\beta\:}_{2g\left(s\right)}*\left(t-2000\right)+{u}_{0c}+{\epsilon\:}_{c,g\left(s\right),t}\:\left[1-Hypotheses,\:\alpha\:=0.05\right]$$

In the multilevel GLMMs constructed for this study, the effects of the development level and time (year) on FLP were tested. The following hypotheses are formulated and evaluated:

- **H₀₁**: No significant difference in FLP exists between developing and developed countries *(β*_*1label*_ *= 0)*.
- **H₁₁**: A significant difference in FLP exists between developing and developed countries *(β*_*1label*_ *≠ 0)*.
- **H₀₂**: Time (year) has no significant effect on FLP *(β*_*2year*_ *= 0)*.
- **H₁₂**: Time (year) has a significant effect on FLP *(β*_*2year*_ *≠ 0)*.

For the fixed effects in the models, the estimated coefficients (β), standard errors, *t*-values, and *p*-values were reported. The variance and standard deviation of the random intercepts were also calculated, along with the marginal R² (variance explained by fixed effects) and the conditional R² (variance explained by both fixed and random effects). To strengthen the methodological framework, we incorporated a sensitivity analysis of the exponential temporal weighting parameter, testing values across the range of 0.00–0.50. This procedure confirmed λ = 0.20 as the optimal configuration for maximizing cluster separation (while maintaining high classification stability (> 98% agreement). Notably, this analysis identified two countries—New Zealand and the Republic of Korea—that exhibited borderline shifts between developed and developing clusters under alternative λ values. These cases were subsequently highlighted with descriptive statistics in the results as examples of transitional economic profiles. All analyses were performed using the R programming language (version 4.4.2). Visualizations were created using the *ggplot2* package, clustering was conducted with the *cluster* package, and GLMM modeling was performed using the *lme4* package^29–31^.

## Results

This section presents the results of multilevel analyses and temporal trends that reveal differences in FLPs across countries by development level, commodity groups, and supply chain stages.

First, countries were classified into “developed” and “developing” categories using a K-means clustering approach based on three core socioeconomic indicators: GDP per capita, GDP growth rate, and health expenditure per capita. To account for the diminishing influence of historical data, a temporal weighting function was applied. A sensitivity analysis across λ values (0.00–0.50, incremented by 0.05) was conducted to determine the optimal weighting parameter and assess classification stability (Table 1).

**Table 1:** Sensitivity of country classification to Temporal weighting (λ).

Lambda	Silhouette Score	Suggested optimal k	Silhouette for suggested optimal k	Developed (*n*)	Developing (*n*)
0.00	0.6849006	2	0.6849006	12	93
0.05	0.6819273	2	0.6819273	13	92
0.10	0.6865926	2	0.6865926	13	92
0.15	0.6924942	2	0.6924942	13	92
**0.20**	**0.6987467**	**2**	**0.6987467**	**13**	**92**
0.25	0.6801862	3	0.7161577	13	92
0.30	0.6818329	3	0.7212277	13	92
0.35	0.6877317	3	0.7246317	13	92
0.40	0.687781	3	0.7224686	14	91
0.45	0.6880869	3	0.7251968	14	91
0.50	0.694156	3	0.7270374	14	91

**Lambda**	**Agreement Rate**	Countries Changed*	Developed Changed to Developing*	Developing Changed to Developed*	
0.00	0.99	1	1	0	
0.05	1.00	0	0	0	
0.10	1.00	0	0	0	
0.15	1.00	0	0	0	
0.25	1.00	0	0	0	
0.30	1.00	0	0	0	
0.35	1.00	0	0	0	
0.40	0.99	1	0	1	
0.45	0.99	1	0	1	
0.50	0.99	1	0	1	

**Country**	**λ = 0**	**λ = 0.05–0.35**	**λ = 0.4–0.5**	**Economic Rationale**	
New Zealand	Developing	Developed	Developed	High GDP; moderate health spending affects weighting	
Republic of Korea	Developing	Developing	Developed	Recent rapid growth accentuated at higher λ	
All Others	Stable	Stable	Stable	Robust classification across weighting range	

* Reclassification relative to λ = 0.20 (highest silhouette score at k = 2).

Silhouette scores generally increased with higher λ values, reflecting improved cluster separation. However, models with λ ≥ 0.25 consistently identified three clusters rather than the intended two, deviating from the theoretical objective of distinguishing “developed” and “developing” groups. Among the two-cluster solutions, λ = 0.20 achieved the highest silhouette score (0.699) and was therefore selected as the optimal weighting parameter for subsequent analyses. Three-cluster solutions were not retained due to the small size of the additional subgroup, its lack of clear socioeconomic distinctiveness, and concerns regarding model interpretability and statistical power. Furthermore, although k = 3 models exhibited slightly lower AIC values relative to k = 2, these differences were marginal and did not warrant the added complexity, particularly given the study’s conceptual emphasis on a binary classification framework.

Classification stability remained high across the sensitivity range, with 103 of 105 countries (98.1%) retaining their group membership. Only two countries exhibited borderline shifts. New Zealand transitioned from “developing” to “developed” at λ = 0.05, likely due to its high GDP per capita relative to moderate health spending. Republic of Korea shifted at λ ≥ 0.40, reflecting the growing influence of its recent economic and health investments under stronger temporal weighting. Together, these findings underscore both the robustness of the clustering procedure and the empirical rationale for adopting λ = 0.20 as the final weighting parameter.

**Fig. 1:**
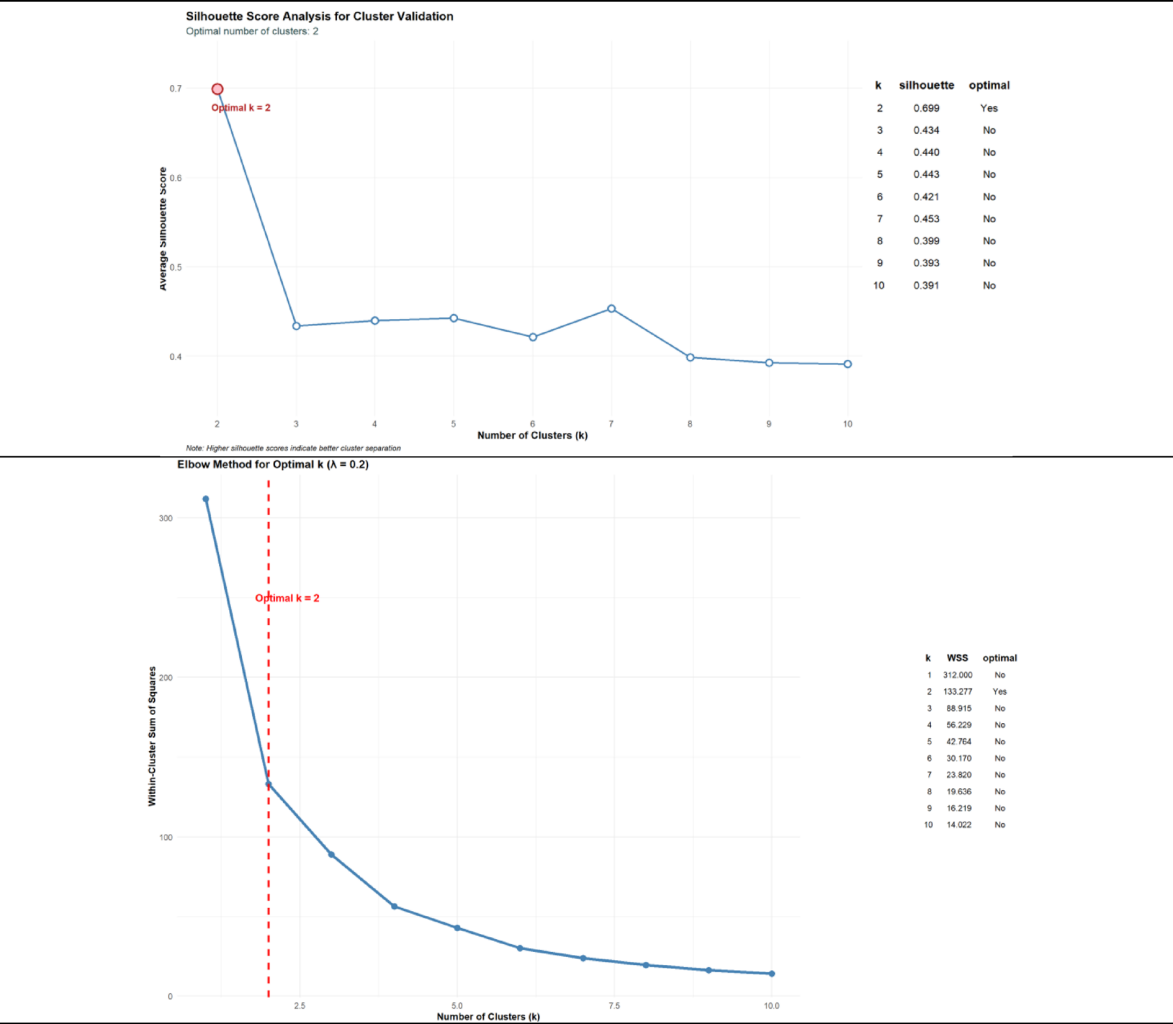
Silhouette Coefficient Analysis for K-means Clustering Validation.

Sensitivity analysis of the temporal-weighting parameter (λ) confirmed that the specification of λ = 0.20 yields a robust and conceptually coherent two-cluster developmental typology. At this setting, the average silhouette score peaked at 0.699 for k = 2 and declined sharply with additional clusters (k = 3: 0.434; k = 4: 0.440), indicating a rapid loss of between-cluster distinctiveness beyond the intended “developed–developing” dichotomy. Complementary evidence from the Elbow method (Fig. 1) reinforces this conclusion. The largest reduction in within-cluster sum of squares (ΔWSS = 178.7) occurred between k = 1 and k = 2, while subsequent decreases were progressively smaller (k = 2→3: ΔWSS = 44.4; k = 3→4: ΔWSS = 32.7), reflecting diminishing marginal returns. Although k ≥ 3 models offered marginal improvements in WSS and slightly lower AIC values relative to k = 2, these gains were insufficient to justify the additional complexity or the reduced interpretability of a three-cluster solution. Retaining λ = 0.20 as the final weighting parameter was thus both theoretically motivated and empirically validated: it preserves the binary classification framework, accommodates the diminishing relevance of older GDP, GDP growth, and health expenditure data, and maintains high classification stability, with 98.1% of countries retaining their group assignments across the sensitivity range. Only New Zealand and Republic of Korea displayed borderline shifts at higher λ values, further underscoring the overall robustness of the clustering structure.

**Fig. 2:**
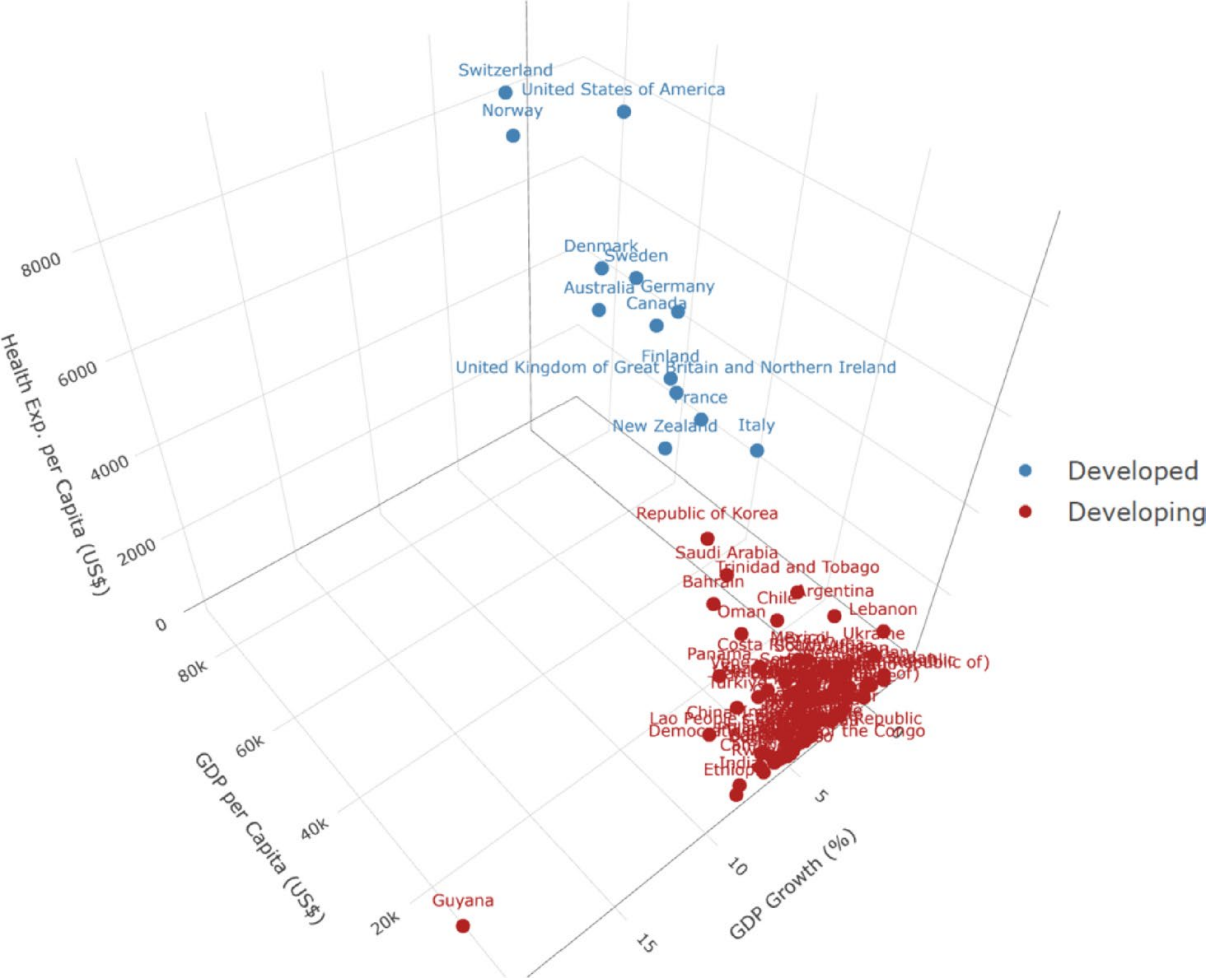
Three-Dimensional Distribution pf Countries Classified as “Developed” or “Developing” Based on GDP Per Capita, GDP Growth Rate, And Health Expenditure Per Capita (Λ = 0.2).

Countries were classified into “developed” (blue) and “developing” (red) groups using a K-means clustering algorithm (k = 2) applied to temporally weighted averages of GDP per capita, GDP growth rate, and health expenditure per capita. The developed cluster (13 countries) exhibits substantially higher socioeconomic indicators, with an average GDP per capita of USD 55,005, health expenditure per capita of USD 5,540, and GDP growth rate of 1.58%. The developing cluster (92 countries) displays lower corresponding values (USD 4,869, USD 259, and 3.19%, respectively). Notably, New Zealand and Republic of Korea occupy borderline positions within their respective clusters. New Zealand, though classified as developed, exhibits comparatively moderate health expenditure relative to its high GDP per capita. Republic of Korea remains in the developing group but approaches the developed threshold, reflecting its recent economic and healthcare advancements. These hybrid cases provide nuanced insights into the transitional dynamics between development categories (Fig. 2).

Following the sensitivity analysis and clustering procedure, the dataset was restructured to include countries, years, commodity types, loss percentages, supply chain stages, and development status. Data were organized in long format, with each observation corresponding to a unique combination of country, year, commodity, and supply chain activity. Initial screening revealed 145 distinct agricultural and food commodities and 125 unique activity types, which were systematically categorized into broader analytical groups. Commodities were classified into 11 major groups: grains, roots and tubers, vegetables, fruits, pulses, meat products, dairy and eggs, oilseeds, sugar products, beverages, and others. Following comprehensive missing data analysis (*detailed in Supplementary Material*,* Missing Value Analysis Section*), ten commodity groups met minimum data requirements for robust statistical modeling: roots and tubers, grains, oilseeds, vegetables, fruits, pulses, sugar products, meat products, dairy and eggs, and other commodities. One group (beverages) was excluded due to insufficient observations for reliable parameter estimation. The retained commodity groups collectively represented 99.9% of available food loss observations, ensuring comprehensive coverage while maintaining analytical rigor. Activity types were categorized into eight main groups—harvest and on-farm operations, processing, transport and distribution, storage, marketing and retail, consumption, packaging, and others—all of which possessed sufficient data for inclusion in the analysis.

**Table 2:** Effects of development status and Temporal trends on food loss percentage by commodity Group.

Commodity Group	*N* (Observations)	*N* (Countries)	Random Intercept (SD)	β	SE	t-value	*p*-value	Marginal *R* ^2^	Conditional *R* ^2^
Intercept				13.353	3.431				
Roots and Tubers	452	33	5.448	1.154	3.345	0.345	0.730	0.018	0.338
**I**_**(Year−2000)**_				**−0.314**	**0.120**	**−2.620**	0.009**		
Intercept				14.117	2.861				
**Grains**	19,585	76	6.854	**−8.023**	**2.977**	**−2.695**	0.007**	0.003	0.864
I_(Year−2000)_				0.005	0.003	1.581	0.114		
Intercept				20.404	7.226				
**Oilseeds**	264	11	10.011	**−19.292**	**7.954**	**−2.426**	0.016*	0.164	0.897
**I**_**(Year−2000)**_				**0.264**	**0.063**	**4.198**	< 0.001***		
Intercept				13.098	2.701				
Vegetables	1462	42	7.088	1.842	2.960	0.622	0.534	0.007	0.420
I_(Year−2000)_				0.032	0.052	0.620	0.536		
Intercept				10.351	3.443				
Fruits	1091	42	6.588	3.619	3.610	1.002	0.316	0.025	0.382
I_(Year−2000)_				−0.048	0.065	−0.735	0.463		
Intercept				13.580	2.740				
**Pulses**	256	24	3.743	**−5.434**	**2.333**	**−2.329**	0.021*	0.061	0.262
I_(Year−2000)_				0.034	0.139	0.245	0.807		
Intercept				5.533	4.576				
Sugar	38	5	7.128	2.925	6.772	0.432	0.669	0.042	0.874
**I**_**(Year−2000)**_				**0.260**	**0.126**	**2.058**	0.048*		
Intercept				5.231	4.898				
Meat	97	14	8.583	0.115	5.407	0.021	0.983	0.009	0.764
I_(Year−2000)_				0.228	0.148	1.545	0.126		
Intercept				13.872	3.749				
Dairy and eggs	33	9	3.062	2.387	2.614	0.913	0.369	0.210	0.598
**I**_**(Year−2000)**_				**−0.893**	**0.294**	**−3.034**	0.005**		
Intercept				13.922	3.554				
Other	1033	41	8.285	0.528	3.921	0.135	0.893	0.000	0.376
I_(Year−2000)_				−0.022	0.069	−0.323	0.747		

Maximum Likelihood; Generalized Liner Mixed Model; Reference Category: Developed; *: *p* < 0.05; **: *p* < 0.01; ***: *p* < 0.001.

Significant differences in FLPs were observed across certain commodity groups based on the countries’ levels of development. Developing countries exhibited significantly lower loss rates in *grains* (β_(label)_ = − 8.023; *p* = 0.007), *oilseeds* (β_(label)_ = − 19.292; *p* = 0.016), and *pulses* (β_(label)_ = − 5.434; *p* = 0.021), whereas the effect of the development level was not statistically significant (*p* > 0.05) for the remaining commodity groups. In terms of temporal trends, a significant increase in FLPs over time was observed in *oilseeds* (β_(year)_ = 0.264; *p* < 0.001) and *sugar* (β_(year)_ = 0.260; *p* = 0.048). In contrast, a significant decline over time was identified in *roots & tubers* (β_(year)_ = − 0.314; *p* = 0.009) and in *dairy & eggs* (β_(year)_ = − 0.893; *p* = 0.005). No statistically significant year effects were found in the other groups. The marginal R² values indicate that the variance explained jointly by development status and year is generally limited (typically < 10%), suggesting these fixed effects account for only a small portion of the total FLP variance. In contrast, conditional R² values were substantially higher across many commodity groups (ranging from 0.26 to 0.90), indicating that random effects at the country level (i.e., persistent between-country differences) explain a large share of the variability. The standard deviations of the random intercepts ranged from 3.06 to 10.01, reflecting the extent of cross-country variation in FLPs (Table 2).

**Table 3:** Effects of development status and Temporal trends on food loss percentage by activity Group.

Activity Group	*N* (Observations)	*N* (Countries)	Random Intercept (SD)	β	SE	t-value	*p*-value	Marginal *R* ^2^	Conditional *R* ^2^
Intercept				8.836	3.866				
Storage	5758	71	9.367	−1.652	4.040	−0.409	0.683	0.000	0.846
I_(Year−2000)_				−0.007	0.009	−0.822	0.411		
Intercept				7.769	1.980				
Harvest and On-Farm	8692	74	5.426	−1.768	2.093	−0.845	0.398	0.001	0.777
I_(Year−2000)_				−0.001	0.005	−0.254	0.800		
Intercept				1.476	3.540				
Transport and Distribution	5082	57	3.431	1.357	3.570	0.380	0.704	0.000	0.838
I_(Year−2000)_				0.002	0.003	0.476	0.634		
Intercept				10.323	5.638				
Packaging	174	14	7.334	−1.647	5.878	−0.280	0.780	0.008	0.751
I_(Year−2000)_				0.092	0.095	0.967	0.335		
Intercept				16.838	4.386				
**Consumption**	174	14	2.697	**−16.056**	**2.949**	**−5.445**	< 0.001***	0.321	0.380
I_(Year−2000)_				0.413	0.382	1.081	0.281		
Intercept				10.231	2.819				
**Processing**	210	26	3.258	**−5.581**	**2.247**	**−2.483**	0.014*	0.063	0.288
I_(Year−2000)_				0.005	0.146	0.034	0.973		
Intercept				7.338	3.436				
Marketing and Retail	613	33	5.306	−0.935	3.414	−0.274	0.784	0.025	0.323
**I**_**(Year−2000)**_				**0.251**	**0.088**	**2.840**	0.005**		
Intercept				13.481	2.310				
Other	3611	90	6.660	−3.641	2.416	−1.507	0.132	0.027	0.393
I_(Year−2000)_				−0.029	0.029	−1.014	0.310		

Maximum Likelihood; Generalized Liner Mixed Model; Reference Category: Developed; *: *p* < 0.05; **: *p* < 0.01; ***: *p* < 0.001.

The effects of development level on FLP by supply chain stage were found to be significantly lower in developing countries than developed countries in the consumption (β_(label)_ = − 16.056; *p* < 0.001) and processing (β_(label)_ = − 5.581; *p* = 0.014) stages. No statistically significant differences were observed for the other stages (*p* > 0.05). In terms of temporal trends, the marketing and retail stage was the only category in which the year variable was statistically significant (β_(year)_ = 0.251; *p* = 0.005), indicating a meaningful upward trend in FLW over time in this stage. While the year variable was not significant for the remaining stages, minor positive or negative tendencies were observed in some cases (*p* > 0.05). Marginal R² values (reflecting variance explained by development level and year combined) were generally low across most stages (ranging from 0.0 to 6.3%), with the exception of the consumption stage, which showed a notably higher explanatory power at 32.1%. This suggests that development level and temporal trends are particularly relevant determinants of the FLP during the consumption phase. Conditional R² values (total variance explained including country-level random effects) were relatively high across many stages, ranging from 0.288 to 0.846, indicating that cross-country variation explains a substantial portion of the variability in food loss. The standard deviations of the random intercepts varied between 2.70 and 9.37, underscoring the considerable differences between countries across supply chain stages (Table 3).

**Fig. 3:**
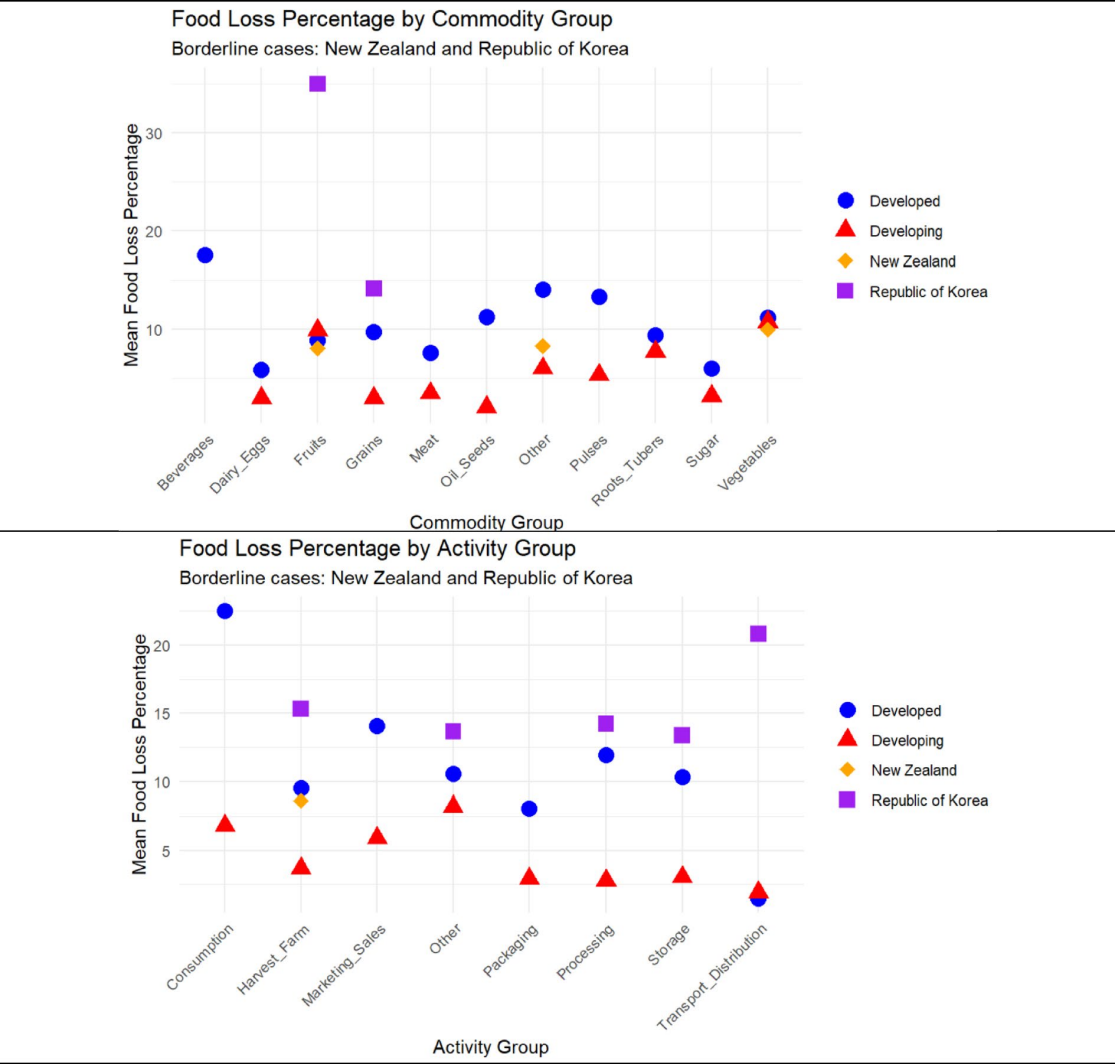
Mean Food-loss Percentage by Commodity and Activity Groups for Developed, Developing, and Borderlines.

**Fig. 4:**
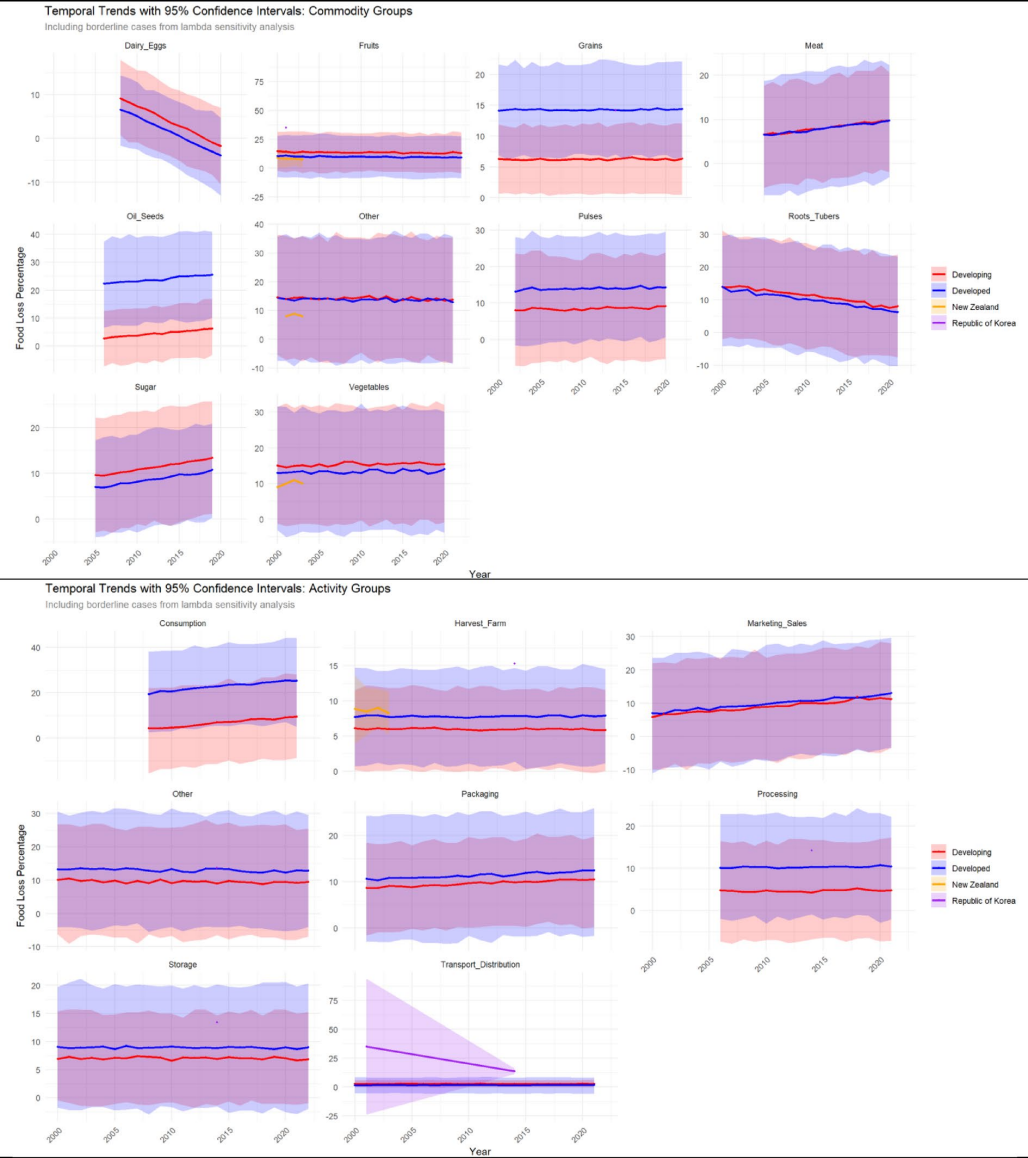
Temporal Trends in FLP by Commodity and Supply Chain Stage Across Developmental Groups and Borderline Cases (2000–2022).

Figure 3 illustrates cross-sectional averages of food loss percentages (FLPs) aggregated across all available years (2000–2022), offering a static snapshot of commodity- and activity-level differences between developed, developing, and borderline countries. Developed nations consistently display higher mean FLPs at later supply chain stages—particularly consumption (22.5%) and beverages (17.6%)—highlighting downstream inefficiencies typical of high-income economies. Conversely, developing countries’ losses are concentrated in upstream stages such as harvest and farm operations (3.7%) and packaging (3.2%), with overall lower FLPs across most commodities. New Zealand’s data (*n* = 15; 2000–2003) show intermediate values: harvest/farm losses averaging 8.6% and commodity-level FLPs (fruits: 8.1%; vegetables: 10.0%) falling between developed (mean = 11.5%) and developing (3.6%) benchmarks. Korea’s sparse but more recent data (*n* = 35; 2001, 2014) reveal exceptionally high mean losses across multiple activities (e.g., transport/distribution: 20.8%) and commodities (fruits: 35%), positioning it closer to developed countries in certain dimensions but with substantial variability. These static averages provide an important reference point for interpreting temporal dynamics in Fig. 4.

For commodity groups, developed countries exhibited statistically significant increasing trends in oil seeds (β = 1.37 ± 0.40, *p* < 0.001) and significant decreasing trends in dairy and eggs (β = −1.13 ± 0.34, *p* < 0.01), while developing countries showed significant decreasing trends in oil seeds (β = −1.13 ± 0.41, *p* < 0.01). No other commodity groups demonstrated statistically significant temporal changes. Loss rates in developed countries generally exhibited greater fluctuations and reached notably high levels in certain years, particularly for vegetables and oil seeds, with wider confidence intervals indicating higher uncertainty in these estimates compared to more stable patterns observed in developing countries. For supply chain stages, several significant temporal trends emerged with distinct patterns between country groups. In developed countries, harvest and farm operations showed significant increasing trends (β = 0.30 ± 0.15, *p* < 0.05), packaging activities demonstrated significant decreasing trends (β = −1.44 ± 0.42, *p* < 0.001), processing exhibited significant decreasing trends (β = −4.56 ± 1.62, *p* < 0.01), and marketing and sales activities showed significant increasing trends (β = 0.78 ± 0.22, *p* < 0.001). Developing countries displayed contrasting patterns with significant increasing trends in packaging (β = 1.61 ± 0.43, *p* < 0.001) and processing (β = 4.62 ± 1.62, *p* < 0.01), alongside decreasing trends in harvest and farm operations (β = −0.30 ± 0.15, *p* < 0.05) and marketing and sales (β = −0.62 ± 0.24, *p* < 0.05) (Fig. 4).

Borderline cases warrant cautious interpretation due to limited temporal data but provide valuable context for the weighting parameter (λ) selection. New Zealand’s dataset (*n* = 15; 2000–2003) covers three commodity groups and exclusively harvest/farm activities, with annual mean FLPs ranging narrowly between 8.3% and 9.0%—consistent with a high-income country experiencing moderate upstream inefficiencies. The Republic of Korea’s dataset (*n* = 35) is skewed toward 2014 (*n* = 33), exhibiting high losses across five activity groups (e.g., transport/distribution: 20.8%) and grains (mean FLP: 14.2%). These elevated values—exceeding both developed (10.2%) and developing (2.7%) group means in 2014—reflect a snapshot of rapid economic and supply chain transformations. However, the uneven temporal coverage (New Zealand: 4 years, 100% coverage; Korea: 14 years, 14.3% coverage) limits robust trend modeling. New Zealand’s consistent moderate loss rates reflect stable, high-income agriculture, whereas Korea’s recent high losses highlight rapid supply chain shifts (Fig. 4).

## Discussion

In developed countries, the highest concentration of FLW occurs at the consumption stage, with a loss rate of 22.5%—markedly higher than in developing countries (6.8%), as well as in transitional economies like New Zealand (9.0%) and the Republic of Korea (14.2%), which exhibit hybrid loss patterns.This striking disparity highlights the significant impact of consumer behavior, portion sizes, food labeling practices, and purchasing habits on food waste in developed contexts. Chrisendo et al.^17^ emphasized that FLW in developed countries largely stems from misconceptions about expiration dates and a tendency toward overconsumption. Their study accentuates the widespread nature of these behaviors and emphasizes the importance of promoting conscious consumption habits and strengthening consumer education programs as key strategies for reducing food waste in developed countries.

Heng and House^32^ compared food waste behaviors among consumers in the United States, Canada, the United Kingdom, and France. The authors integrated portion size, shopping habits, and food labeling with perceptual, behavioral, cultural, and economic dimensions, emphasizing that high-income levels, product abundance, and purchasing power may render consumers less sensitive to food waste. Our findings similarly reveal that developed countries exhibit significantly higher food loss percentages (FLPs) at the consumption stage (22.5%), supporting this hypothesis that affluence and consumer abundance lead to greater waste. This pattern in our study may also reflect systemic issues such as oversized portion norms, weak incentives for household conservation, and limited policy interventions targeting consumer-level waste in high-income settings.

Similarly, in a study of European countries, Tkáč et al.^33^ found that household food waste also rises as GDP per capita increases. They argued that high-income groups, having easier access to food and less economic concern, tend to become less sensitive to food waste. This is consistent with our finding that grains, oilseeds, and pulses in developing countries have significantly lower FLPs than in developed countries, suggesting that scarcity and lower purchasing power may promote more conservative consumption patterns. These differences may also stem from stronger cultural practices of resourcefulness in less affluent economies. These findings are further supported by a comprehensive meta-analysis by Hermanussen and Loy^34^. Their analysis revealed that household-focused studies consistently reported an increase in food waste as income increased. In contrast, higher education levels were statistically associated with a significant reduction in food waste. Interestingly, even self-reported data from survey-based studies showed high levels of waste, although the meta-analysis indicated that observational measurements generally yielded lower waste rates than survey responses. The researchers stressed that education and cognitive awareness are variables independent of economic prosperity and they strongly advocated for education-based interventions to effectively reduce food waste. This resonates with our result that the consumption stage’s FLP is particularly sensitive to development status, where educational initiatives aimed at increasing consumer awareness could potentially mitigate these losses. Given that our analysis also shows significant declines over time in perishables such as dairy and eggs in developed countries, this may reflect the success of targeted educational campaigns and infrastructural improvements such as cold chains. Adopting a different approach, a recent time series analysis highlighted the insufficient linkage between educational investment and FLW outcomes, arguing that sustainability principles should be more central to formal education curricula^35^. Our finding that temporal trends in some commodity groups (e.g., oilseeds and sugar) are increasing even in developed countries suggests that educational investments alone may not suffice unless coupled with structural and policy interventions. This underscores the need for integrating sustainability deeply into curricula and complementing it with systemic changes such as improved food labeling and portion control regulations. Collectively, these findings demonstrate that food waste is influenced not only by individual consumer choices but also by national levels of economic prosperity, investments in education, and policy priorities. In line with this, our results reinforce the importance of combining consumer-level interventions with broader systemic reforms to address the disproportionately high consumption-stage losses in developed economies.

Treated as control variables, the study’s temporal trends revealed a significant increase over time in the oilseed and sugar groups, whereas a significant decline was observed in the roots and tubers and dairy and eggs groups. The decline in perishables, such as dairy and eggs, may be attributed to expanding cold chain systems, while including root and tuber crops in short supply chains may reduce losses. In contrast, the increasing trend in oilseeds and sugar may reflect the nature of industrial-scale production and bioenergy demand, and the greater risk of consumer-level waste associated with products with extended shelf lives. Consistent with these our findings, Durán-Sandoval, Durán-Romero, and Uleri^36^ reported that technological improvements and supply chain enhancements helped reduce losses in product groups, such as starchy roots, whereas losses persisted in items vulnerable to consumption-stage waste, including sugary and processed foods. These findings suggest that, in addition to the effects of time and development level, interventions based on treatment and information may also yield positive outcomes in reducing food loss. Several studies have shown that intervention programs that emphasize healthy eating habits can increase fruit and vegetable consumption among youth^37,38^. Moreover, promoting healthy nutrition not only contributes to individual well-being but also improves public health outcomes^37^. When contextualized within local cultures and traditions, country-level differences provide a broader perspective on FLW.

In our analysis, the level of development and time jointly explained 32.1% of the variance in food loss at the consumption stage (Marginal R² = 0.321), the highest among all activity groups. This finding reveals a critical insight that contrasts with many previous assumptions: the consumption process is highly responsive to structural factors and can be effectively reacted to. A comparative study of India and the Netherlands found that food waste is heavily influenced by structural and social factors, such as consumer behavior, cultural norms, and food labeling literacy, and may be effectively reduced through digital solutions^39^. This underscores the importance of developing consumer-oriented interventions (e.g., digital food-sharing platforms, labeling reforms, awareness campaigns) not only to shape behavior but also as policy-sensitive and systemic strategies to deliver widespread impact. Moreover, our GLMM models indicated that when country-level random effects were included, the conditional R² rose to 84.6%, underscoring that intercountry differences are the leading determinants of food loss. More than a technical outcome, this indicates a deeper structural reality: unless control mechanisms, such as educational spending, in developed countries are mirrored in developing nations through publicly funded, targeted, and capacity-enhancing policies, structural inequalities in food loss may persist. A case study of Spain’s Catalonia region analyzed the economic and social impacts of food loss in the vegetable sector at spatial and temporal scales, identifying summer losses in watermelons, tomatoes, and lettuce as high as 41% in Tarragona and 34% in Barcelona. These losses were frequently the result of consumers discarding edible products due to cosmetic imperfections that contradict local esthetic norms^40^. These examples suggest that national-level differences are not merely the result of technical inefficiencies but are also rooted in consumer perceptions, market standards, and social norms. Thus, many drivers of food loss may be structural yet modifiable and must be addressed in evidence-based policy development.

Although our study found no statistically significant differences in food loss at the marketing and retail stages by country or development level, a significant increase in FLW was observed over time in these stages. A study conducted in Ukraine reported that FLW occurred throughout the entire supply chain, with annual per capita food waste exceeding 250 kg; 95% of this waste was landfilled, posing a serious risk to environmental sustainability^41^. These findings suggest that in developed countries, the high demand for fresh products and uncertainty surrounding “best before” dates contribute to elevated waste rates. In contrast, in developing countries, infrastructure deficiencies and logistical challenges often result in higher losses during the early stages of the supply chain. Rahman et al.^42^ noted that advanced recycling and regulatory systems in Taiwan have contributed significantly to reducing food waste, whereas in countries like Bangladesh, regulatory challenges and limited technological infrastructure have exacerbated FLW. A recent bibliometric analysis by Wang, Morkūnas, and Wei^43^ found that agricultural practices targeting food loss reduction and climate change adaptation can enhance efficiency in the food supply chain while simultaneously mitigating environmental impacts.

Countries with hybrid development profiles—defined by mid-level socioeconomic indicators, transitional economic structures, and uneven institutional capacities—exhibit complex, heterogeneous food loss and waste patterns across the supply chain. Clustering on GDP, economic momentum, and health spending positions New Zealand and the Republic of Korea as boundary cases: New Zealand, despite its stable high-income status, records upstream food loss of about 8.6% at harvest and on-farm stages, squarely between developed (11.5%) and developing (3.6%) benchmarks, largely due to stringent cosmetic-quality standards, overproduction incentives, and inflexible commodity grading that encourage culling of edible produce; Reynolds et al.^13^ show these losses concentrate in processed foods and embed heavy environmental footprints (≈ 4.2 Mt CO₂-e and 4.7 Gm³ water annually), while Goodman-Smith et al.^11^ trace significant downstream retail losses to tight quality thresholds and logistics frictions, implying the need for more flexible cosmetic tolerances, loss-sharing clauses in producer–retailer contracts, and demand-driven inventory platforms. Conversely, Korea demonstrates a more dynamic yet imbalanced hybrid scenario, with food loss and waste rates that surpass developed-country averages (20.8% i transport/distribution and 35% i fruit), a by-product of rapid GDP growth, urbanization, and retail modernization; Kim & Park^10^ these figures to fragmented supply-chain coordination, inadequate cold-chain capacity, and policy gaps, suggesting that integrated public–private cold-chain investment programs, real-time digital traceability systems, and hub-and-spoke regional logistics centers could simultaneously curb losses and sustain growth momentum. These cases powerfully illustrate that tailored interventions are essential for addressing both upstream infrastructure deficiencies and downstream regulatory-logistics challenges. When standardized data systems, regionally adapted fiscal incentives (such as “loss-pays” mechanisms), and cross-border knowledge sharing networks work together, food loss mitigation transforms from scattered pilot projects into a comprehensive, integrated system that generates widespread global benefits.

### Limitations

This study is subject to several limitations stemming primarily from its reliance on secondary data sources. Although data were acquired from reputable international institutions such as the World Bank and FAO, methodological discrepancies, differing reporting standards, and institutional capacities across countries may compromise data comparability and quality. The observational nature of our analysis, despite incorporating time as an auxiliary variable, inherently constrains our ability to robustly capture temporal dynamics and thus provides limited evidence for causal inferences. Additionally, the necessity of listwise deletion due to missing observations further restricts representativeness and might obscure important effects. While temporal influences were addressed both directly through a fixed annual control and indirectly via cumulative contributions from GDP, economic momentum, and health expenditures, this dual approach remains insufficient to fully encapsulate policy shocks, technological advancements, or other dynamic temporal effects. The weighting scheme itself relies on assumptions whose sensitivity to alternative λ values was partly illustrated through cases such as Korea and New Zealand, underscoring another methodological limitation only partially mitigated through descriptive assessments. Furthermore, our clustering method, which segments countries strictly based on economic and health indicators, risks oversimplifying complex developmental realities—particularly masking heterogeneity among hybrid economies at the boundary between developed and developing status. While these hybrid cases were descriptively explored, future research should explicitly integrate structural and institutional variables to refine the analytical depth. Importantly, the selected economic and health predictors omit critical determinants like agricultural infrastructure, logistical capacities, and consumer behavior patterns, thereby limiting the comprehensive identification of multidimensional drivers behind food loss percentages. It is also critical to acknowledge potential endogeneity concerns, as independent variables may simultaneously act as outcomes, influencing each other through complex bidirectional interactions. Future investigations could employ causal modeling frameworks such as structural variance autoregressive models or instrumental variable approaches utilizing instruments like climatic factors (e.g., rainfall variability, temperature anomalies), political stability indices, or agricultural policy indicators (e.g., subsidies, import/export restrictions) to strengthen causal interpretations. Ultimately, richer country-level datasets coupled with dynamic econometric methods, such as panel data or time-series analyses, will be necessary to overcome these constraints and enable more robust causal modeling. Despite these limitations, given the currently limited research in this area, we believe this study provides a valuable starting point and foundational insights for future exploration.

## Conclusions and recommendations

Developed economies predominantly experience significant losses at the consumption stage, while developing economies face substantial challenges upstream, particularly during harvesting and on-farm operations. The significant explanatory power of country-specific random effects in our models underscores the necessity of context-specific interventions tailored to local conditions rather than generic global prescriptions. Firstly, for all countries, it is essential to prioritize large-scale consumer awareness campaigns promoting portion control, clearly distinguishing between “use by” and “best before” dates, and normalizing surplus food redistribution, as our finding that the consumption stage explains approximately one-third of the variance in food loss highlights the importance of consumer-focused interventions. Moreover, implementing regulatory mandates such as “surplus-to-sale” or “surplus-to-donation” for retailers—backed by fiscal incentives for compliance and escalating taxes on avoidable waste—can substantially reduce downstream waste. Additionally, enhancing traceability through alignment with SDG 12.3 indicators, employing targeted economic tools like subsidies for efficient practices, and removing legal barriers to food banking constitute critical steps toward universal adoption.

For developed countries, interventions should specifically target consumer behaviors, emphasizing policies designed to regulate portion sizes, reform labeling practices, and conduct educational campaigns addressing overconsumption and misconceptions about food waste. These countries should also strengthen regulatory frameworks surrounding retail and marketing practices, such as demand-driven inventory management systems and flexible cosmetic-quality standards. For developing countries, our identification of significantly higher upstream losses—particularly in harvest and on-farm operations—underscores the need for strategic investments in affordable post-harvest technologies, expanded microfinance for mechanization, and climate-resilient regional storage facilities. Nationwide farmer-training initiatives focusing on good agricultural practices, logistics management, and infrastructure improvements will substantially reduce these early-stage losses.

Hybrid economies exemplified by New Zealand and the Republic of Korea require dual-track approaches due to simultaneous upstream and downstream FLW challenges. In Korea, fragmented cold-chain logistics could be addressed effectively through integrated public-private investments, establishment of real-time digital traceability systems, and regional logistics hubs, providing a crucial example of intervention for rapidly growing economies experiencing structural transitions. Concurrently, consumer-focused initiatives that enhance food literacy—especially around portion sizes and expiration dates—are vital. For New Zealand, relaxing cosmetic-quality standards within safety parameters, embedding “loss-sharing” clauses in producer-retailer contracts, and expanding surplus redistribution channels (such as gleaning and secondary markets) represent critical systemic steps toward waste reduction. Such strategies exemplify practical interventions to manage risks associated with countries sharing similar levels of development and regulatory structures.

Ultimately, our findings clearly demonstrate that integrating public institutions, private enterprises, producers, and consumers within comprehensive, multi-stakeholder collaborations tailored specifically to each country’s unique economic and institutional context holds the strongest potential to achieve substantial and lasting reductions in global food loss and waste.

## Supplementary Information

Below is the link to the electronic supplementary material.

Supplementary Material 1

## Data Availability

The dataset generated and analyzed during the current study is openly available in Zenodo at https://doi.org/10.5281/zenodo.15357549.”
