# Supplementary material for: Understanding food loss patterns across developed and developing countries using a GDP, growth rate, and health expenditure-based typology
Source: Sci Rep. 2025 Jul 29;15:27597. doi: 10.1038/s41598-025-13156-3 (PMC12307759; doi:10.1038/s41598-025-13156-3)
Supplement: Supplementary file 1 — Supplementary Material 1 [file 41598_2025_13156_MOESM1_ESM.docx]

**SUPPLEMENTARY CHAPTER 1:**

**MISSING DATA ANALYSIS**

Table S1 shows the extent of missing data across years and key variables. Missing rates for loss percentage are relatively low (typically <7%) from 2000–2021 but rise sharply in 2022–2023 due to limited country and commodity coverage in recent years.

Table S1. Missing rates by year and key variables

| **Year** | **Total Observation** | **Missing loss PCT** | **Missing Loss Rate** | **Available Countries** | **Available Commodities** |
| --- | --- | --- | --- | --- | --- |
| 2000 | 974 | 64 | 6,57 | 41 | 59 |
| 2001 | 1136 | 59 | 5,19 | 46 | 70 |
| 2002 | 1025 | 59 | 5,76 | 46 | 63 |
| 2003 | 993 | 60 | 6,04 | 45 | 64 |
| 2004 | 949 | 63 | 6,64 | 42 | 57 |
| 2005 | 1011 | 58 | 5,74 | 47 | 64 |
| 2006 | 1435 | 64 | 4,46 | 41 | 86 |
| 2007 | 972 | 59 | 6,07 | 46 | 61 |
| 2008 | 1234 | 58 | 4,7 | 47 | 84 |
| 2009 | 1549 | 60 | 3,87 | 45 | 63 |
| 2010 | 1331 | 59 | 4,43 | 46 | 75 |
| 2011 | 1108 | 50 | 4,51 | 55 | 72 |
| 2012 | 1109 | 54 | 4,87 | 51 | 68 |
| 2013 | 1295 | 34 | 2,63 | 71 | 90 |
| 2014 | 1591 | 53 | 3,33 | 52 | 93 |
| 2015 | 1258 | 42 | 3,34 | 63 | 83 |
| 2016 | 1319 | 52 | 3,94 | 53 | 90 |
| 2017 | 1104 | 56 | 5,07 | 49 | 66 |
| 2018 | 1262 | 58 | 4,6 | 47 | 71 |
| 2019 | 1045 | 62 | 5,93 | 43 | 47 |
| 2020 | 907 | 66 | 7,28 | 39 | 21 |
| 2021 | 952 | 65 | 6,83 | 40 | 10 |
| 2022 | 112 | 102 | 91,07 | 3 | 4 |
| 2023 | 105 | 105 | 100 | 0 | 0 |

Table S2 summarizes sample sizes for each commodity and activity group. Grains and Harvest_Farm have the largest number of observations with broad temporal coverage, while groups like Beverages and Consumption are more limited, reflecting uneven data availability across categories.

Table S2. Sample Sizes by Commodity and Activity Groups

| **Commodity Group** | **Observations (n)** | **Countries (n)** | **Years (n)** | **Year Range** |
| --- | --- | --- | --- | --- |
| Grains | 19585 | 76 | 23 | 2000 - 2022 |
| Vegetables | 1462 | 42 | 21 | 2000 - 2020 |
| Fruits | 1091 | 42 | 22 | 2000 - 2022 |
| Other | 1033 | 41 | 22 | 2000 - 2021 |
| Roots_Tubers | 452 | 33 | 22 | 2000 - 2021 |
| Oil_Seeds | 264 | 11 | 12 | 2006 - 2019 |
| Pulses | 256 | 24 | 15 | 2002 - 2020 |
| Meat | 97 | 14 | 15 | 2005 - 2020 |
| Sugar | 38 | 5 | 10 | 2005 - 2019 |
| Dairy_Eggs | 33 | 9 | 12 | 2008 - 2020 |
| Beverages | 3 | 2 | 2 | 2008 - 2020 |
| **Activity Group** | **Observations (n)** | **Countries (n)** | **Years (n)** | **Year Range** |
| Harvest_Farm | 8692 | 74 | 23 | 2000 - 2022 |
| Storage | 5758 | 71 | 23 | 2000 - 2022 |
| Transport_Distribution | 5082 | 57 | 22 | 2000 - 2021 |
| Other | 3611 | 90 | 23 | 2000 - 2022 |
| Marketing_Sales | 613 | 33 | 19 | 2000 - 2021 |
| Processing | 210 | 26 | 14 | 2006 - 2021 |
| Consumption | 174 | 14 | 10 | 2008 - 2021 |
| Packaging | 174 | 14 | 15 | 2001 - 2021 |

Table S3 highlights country-specific missingness rates and temporal coverage. Many countries show high missingness (>75%) and short time spans, while others (e.g., Ethiopia, Uganda, Kenya) have extensive coverage with low missingness. Türkiye’s coverage (4 years) is noted as limited.

Table S3. Country-Level Missingness and Temporal Coverage

| **Country** | **Total Observations** | **Missing Loss PCT** | **Missing Loss Rate** | **Available Years** | **Year Range** |
| --- | --- | --- | --- | --- | --- |
| Algeria | 49 | 48 | 97,96 | 1 | 2005 - 2005 |
| Bahrain | 49 | 48 | 97,96 | 1 | 2005 - 2005 |
| Gabon | 49 | 48 | 97,96 | 1 | 2013 - 2013 |
| Germany | 49 | 48 | 97,96 | 1 | 2011 - 2011 |
| Oman | 49 | 48 | 97,96 | 1 | 2011 - 2011 |
| Paraguay | 50 | 48 | 96 | 1 | 2013 - 2013 |
| Serbia | 49 | 47 | 95,92 | 2 | 2008 - 2009 |
| Canada | 51 | 48 | 94,12 | 1 | 2014 - 2014 |
| Colombia | 51 | 48 | 94,12 | 1 | 2016 - 2016 |
| Nicaragua | 51 | 48 | 94,12 | 1 | 2013 - 2013 |
| Switzerland | 51 | 48 | 94,12 | 1 | 2015 - 2015 |
| Tunisia | 51 | 48 | 94,12 | 1 | 2017 - 2017 |
| Chile | 50 | 47 | 94 | 2 | 2013 - 2015 |
| Costa Rica | 52 | 48 | 92,31 | 1 | 2015 - 2015 |
| Italy | 52 | 48 | 92,31 | 1 | 2020 - 2020 |
| Haiti | 51 | 47 | 92,16 | 2 | 2010 - 2011 |
| Lebanon | 51 | 47 | 92,16 | 2 | 2017 - 2022 |
| Kazakhstan | 53 | 48 | 90,57 | 1 | 2014 - 2014 |
| Venezuela | 53 | 48 | 90,57 | 1 | 2013 - 2013 |
| El Salvador | 52 | 47 | 90,38 | 2 | 2013 - 2016 |
| Guyana | 54 | 48 | 88,89 | 1 | 2015 - 2015 |
| Honduras | 54 | 48 | 88,89 | 1 | 2016 - 2016 |
| Myanmar | 54 | 48 | 88,89 | 1 | 2015 - 2015 |
| Lao People's Democratic Republic | 53 | 47 | 88,68 | 2 | 2008 - 2015 |
| Palestine | 56 | 48 | 85,71 | 1 | 2018 - 2018 |
| Peru | 55 | 47 | 85,45 | 2 | 2013 - 2016 |
| Ecuador | 54 | 46 | 85,19 | 3 | 2012 - 2016 |
| Panama | 56 | 47 | 83,93 | 2 | 2012 - 2013 |
| Trinidad and Tobago | 56 | 47 | 83,93 | 2 | 2013 - 2015 |
| Guatemala | 55 | 46 | 83,64 | 3 | 2012 - 2016 |
| Malaysia | 57 | 47 | 82,46 | 2 | 2007 - 2010 |
| Timor-Leste | 59 | 48 | 81,36 | 1 | 2015 - 2015 |
| France | 60 | 48 | 80 | 1 | 2015 - 2015 |
| Syrian Arab Republic | 59 | 47 | 79,66 | 2 | 2017 - 2018 |
| Argentina | 61 | 48 | 78,69 | 1 | 2019 - 2019 |
| Jordan | 58 | 45 | 77,59 | 4 | 2002 - 2019 |
| Türkiye | 58 | 45 | 77,59 | 4 | 2002 - 2014 |
| Egypt | 57 | 44 | 77,19 | 5 | 2011 - 2017 |
| Viet Nam | 59 | 45 | 76,27 | 4 | 2008 - 2015 |
| Cuba | 64 | 48 | 75 | 1 | 2011 - 2011 |
| New Zealand | 60 | 45 | 75 | 4 | 2000 - 2003 |
| Thailand | 60 | 45 | 75 | 4 | 2007 - 2016 |
| Saudi Arabia | 64 | 47 | 73,44 | 2 | 2015 - 2019 |
| Brazil | 61 | 44 | 72,13 | 5 | 2003 - 2015 |
| Fiji | 65 | 44 | 67,69 | 5 | 2000 - 2012 |
| Denmark | 72 | 45 | 62,5 | 4 | 2011 - 2017 |
| Norway | 72 | 45 | 62,5 | 4 | 2011 - 2017 |
| Indonesia | 71 | 41 | 57,75 | 8 | 2002 - 2013 |
| Republic of Korea | 82 | 47 | 57,32 | 2 | 2001 - 2014 |
| Armenia | 85 | 47 | 55,29 | 2 | 2009 - 2013 |
| Australia | 88 | 45 | 51,14 | 4 | 2013 - 2019 |
| Ukraine | 93 | 47 | 50,54 | 2 | 2013 - 2014 |
| Morocco | 93 | 46 | 49,46 | 3 | 2015 - 2018 |
| Cambodia | 94 | 44 | 46,81 | 5 | 2005 - 2015 |
| Mexico | 96 | 44 | 45,83 | 5 | 2007 - 2016 |
| Sweden | 103 | 45 | 43,69 | 4 | 2011 - 2017 |
| Finland | 101 | 43 | 42,57 | 6 | 2010 - 2017 |
| Sri Lanka | 117 | 46 | 39,32 | 3 | 2002 - 2018 |
| Azerbaijan | 115 | 42 | 36,52 | 7 | 2005 - 2020 |
| Philippines | 119 | 41 | 34,45 | 8 | 2003 - 2022 |
| Nepal | 127 | 37 | 29,13 | 12 | 2000 - 2021 |
| Iran (Islamic Republic of) | 160 | 44 | 27,5 | 5 | 2001 - 2015 |
| United Kingdom | 173 | 45 | 26,01 | 4 | 2011 - 2018 |
| Pakistan | 174 | 37 | 21,26 | 12 | 2001 - 2021 |
| Liberia | 137 | 27 | 19,71 | 22 | 2000 - 2021 |
| China | 253 | 38 | 15,02 | 11 | 2001 - 2021 |
| South Sudan | 181 | 27 | 14,92 | 22 | 2000 - 2021 |
| Eswatini | 182 | 27 | 14,84 | 22 | 2000 - 2021 |
| Madagascar | 256 | 27 | 10,55 | 22 | 2000 - 2021 |
| Sudan | 291 | 27 | 9,28 | 22 | 2000 - 2021 |
| Sierra Leone | 341 | 27 | 7,92 | 22 | 2000 - 2021 |
| Lesotho | 348 | 27 | 7,76 | 22 | 2000 - 2021 |
| Cameroon | 363 | 27 | 7,44 | 22 | 2000 - 2021 |
| Namibia | 379 | 27 | 7,12 | 22 | 2000 - 2021 |
| Bangladesh | 600 | 39 | 6,5 | 10 | 2004 - 2018 |
| South Africa | 422 | 27 | 6,4 | 22 | 2000 - 2021 |
| Somalia | 423 | 27 | 6,38 | 22 | 2000 - 2021 |
| Niger | 427 | 27 | 6,32 | 22 | 2000 - 2021 |
| Botswana | 445 | 27 | 6,07 | 22 | 2000 - 2021 |
| Congo Republic | 445 | 27 | 6,07 | 22 | 2000 - 2021 |
| Guinea | 446 | 27 | 6,05 | 22 | 2000 - 2021 |
| Eritrea | 489 | 27 | 5,52 | 22 | 2000 - 2021 |
| Mauritania | 511 | 27 | 5,28 | 22 | 2000 - 2021 |
| Zimbabwe | 519 | 27 | 5,2 | 22 | 2000 - 2021 |
| Burundi | 533 | 27 | 5,07 | 22 | 2000 - 2021 |
| Chad | 533 | 27 | 5,07 | 22 | 2000 - 2021 |
| Gambia | 556 | 27 | 4,86 | 22 | 2000 - 2021 |
| Guinea-Bissau | 556 | 27 | 4,86 | 22 | 2000 - 2021 |
| Togo | 555 | 27 | 4,86 | 22 | 2000 - 2021 |
| Nigeria | 560 | 27 | 4,82 | 22 | 2000 - 2021 |
| Rwanda | 611 | 27 | 4,42 | 22 | 2000 - 2021 |
| Mozambique | 622 | 27 | 4,34 | 22 | 2000 - 2021 |
| Mali | 644 | 27 | 4,19 | 22 | 2000 - 2021 |
| Benin | 713 | 26 | 3,65 | 23 | 2000 - 2022 |
| Angola | 753 | 27 | 3,59 | 22 | 2000 - 2021 |
| Senegal | 776 | 27 | 3,48 | 22 | 2000 - 2021 |
| Burkina Faso | 783 | 27 | 3,45 | 22 | 2000 - 2021 |
| Malawi | 783 | 27 | 3,45 | 22 | 2000 - 2021 |
| Zambia | 784 | 27 | 3,44 | 22 | 2000 - 2021 |
| Ghana | 810 | 27 | 3,33 | 22 | 2000 - 2021 |
| Uganda | 882 | 27 | 3,06 | 22 | 2000 - 2021 |
| Ethiopia | 1043 | 27 | 2,59 | 22 | 2000 - 2021 |
| India | 1366 | 35 | 2,56 | 14 | 2001 - 2016 |
| Kenya | 1065 | 27 | 2,54 | 22 | 2000 - 2021 |
| United States of America | 1518 | 29 | 1,91 | 20 | 2000 - 2019 |

Table S4 lists commodity-activity combinations with small sample sizes, flagging potential areas of analytical instability. Most cases involve niche commodities or late-stage activities with sparse data points.

Table S4. Commodity-Activity Combinations with Low Sample Size (<50 Observations)

| **Commodity group** | **Activity group** | **Observations (n)** | **Countries (n)** | **Years (n)** |
| --- | --- | --- | --- | --- |
| Beverages | Other | 1 | 1 | 1 |
| Dairy_Eggs | Harvest_Farm | 1 | 1 | 1 |
| Oil_Seeds | Consumption | 1 | 1 | 1 |
| Sugar | Consumption | 1 | 1 | 1 |
| Sugar | Storage | 1 | 1 | 1 |
| Beverages | Consumption | 2 | 1 | 1 |
| Dairy_Eggs | Processing | 2 | 2 | 1 |
| Meat | Packaging | 2 | 1 | 2 |
| Sugar | Packaging | 2 | 1 | 2 |
| Sugar | Processing | 2 | 1 | 2 |
| Dairy_Eggs | Marketing_Sales | 3 | 2 | 2 |
| Meat | Consumption | 3 | 2 | 2 |
| Sugar | Transport_Distribution | 3 | 1 | 3 |
| Dairy_Eggs | Consumption | 4 | 2 | 2 |
| Sugar | Marketing_Sales | 4 | 2 | 3 |
| Meat | Processing | 6 | 4 | 2 |
| Pulses | Processing | 6 | 2 | 5 |
| Meat | Transport_Distribution | 7 | 3 | 5 |
| Pulses | Packaging | 7 | 1 | 4 |
| Meat | Storage | 8 | 2 | 3 |
| Pulses | Consumption | 8 | 3 | 2 |
| Sugar | Other | 8 | 1 | 5 |
| Pulses | Transport_Distribution | 11 | 3 | 5 |
| Meat | Marketing_Sales | 12 | 4 | 3 |
| Roots_Tubers | Packaging | 13 | 5 | 6 |
| Oil_Seeds | Packaging | 15 | 2 | 4 |
| Grains | Consumption | 16 | 5 | 5 |
| Oil_Seeds | Processing | 17 | 4 | 4 |
| Sugar | Harvest_Farm | 17 | 3 | 7 |
| Meat | Harvest_Farm | 18 | 4 | 5 |
| Pulses | Marketing_Sales | 20 | 3 | 7 |
| Fruits | Processing | 21 | 6 | 6 |
| Oil_Seeds | Transport_Distribution | 22 | 4 | 5 |
| Other | Processing | 22 | 6 | 4 |
| Dairy_Eggs | Other | 23 | 7 | 10 |
| Roots_Tubers | Transport_Distribution | 23 | 10 | 9 |
| Roots_Tubers | Consumption | 24 | 7 | 6 |
| Oil_Seeds | Marketing_Sales | 25 | 3 | 4 |
| Vegetables | Processing | 26 | 3 | 4 |
| Other | Packaging | 31 | 4 | 7 |
| Fruits | Packaging | 33 | 8 | 9 |
| Fruits | Consumption | 35 | 4 | 3 |
| Grains | Packaging | 35 | 5 | 8 |
| Vegetables | Packaging | 36 | 7 | 8 |
| Roots_Tubers | Processing | 38 | 8 | 10 |
| Other | Consumption | 40 | 5 | 5 |
| Vegetables | Consumption | 40 | 4 | 4 |
| Meat | Other | 41 | 10 | 13 |
| Other | Transport_Distribution | 41 | 11 | 10 |
| Oil_Seeds | Storage | 42 | 5 | 7 |
| Pulses | Harvest_Farm | 42 | 7 | 11 |
| Vegetables | Transport_Distribution | 45 | 7 | 8 |
| Fruits | Transport_Distribution | 47 | 12 | 15 |


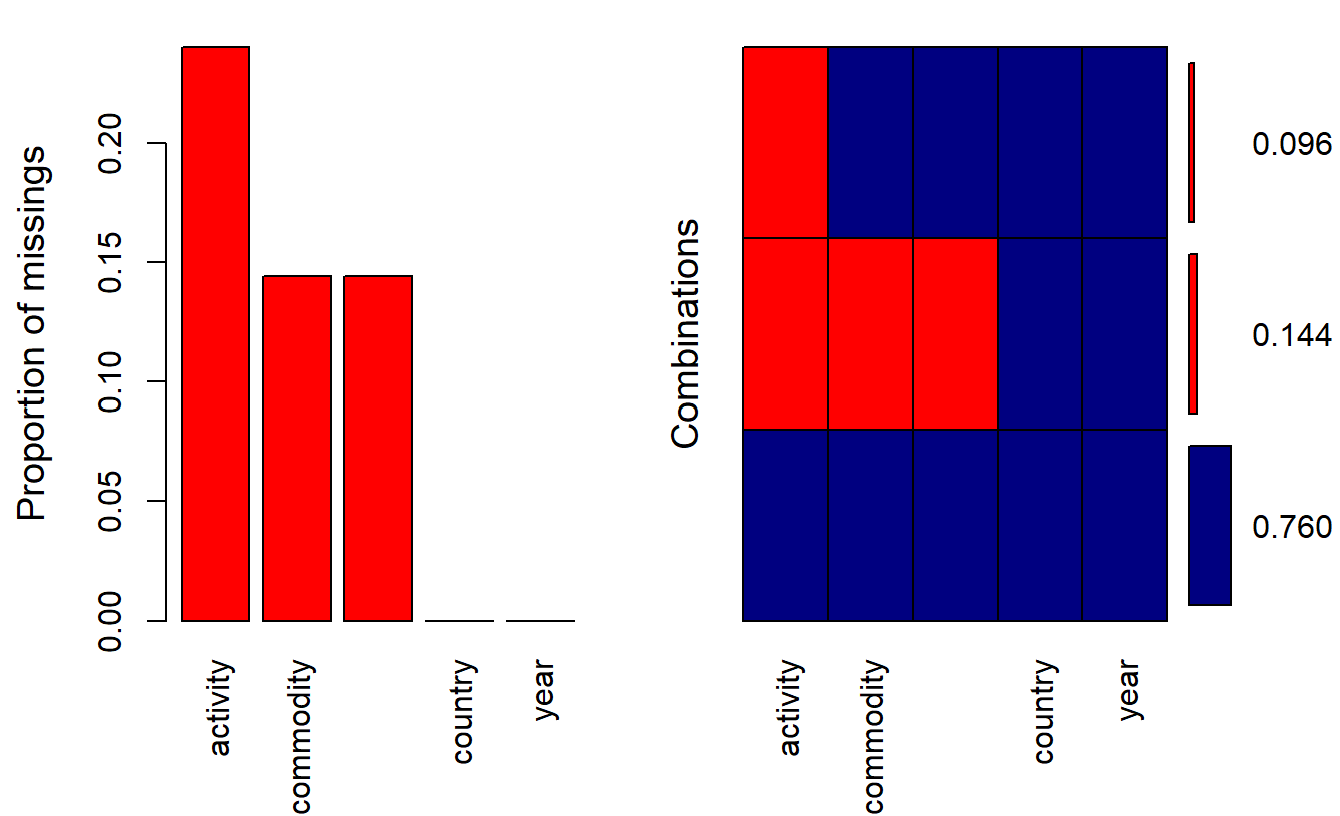


Figure S1. Missing Data Patterns by Variable

Figure presents the proportion and patterns of missing data for key variables in the dataset. The bar chart on the left shows that *activity* has the highest proportion of missing values (~24%), followed by *commodity* and *loss percentage* (~14% each), while *country* and *year* are fully observed. The combination matrix on the right highlights that missingness frequently co-occurs for *activity* and *commodity*, suggesting that data gaps are not randomly distributed but are instead concentrated in specific combinations of variables. Approximately 9.6% of cases are missing only activity, 14.4% are missing both activity and commodity, and 76% have complete data for all key variables. This pattern suggests clustered missingness, indicating structured data gaps rather than random noise (Figure S1).


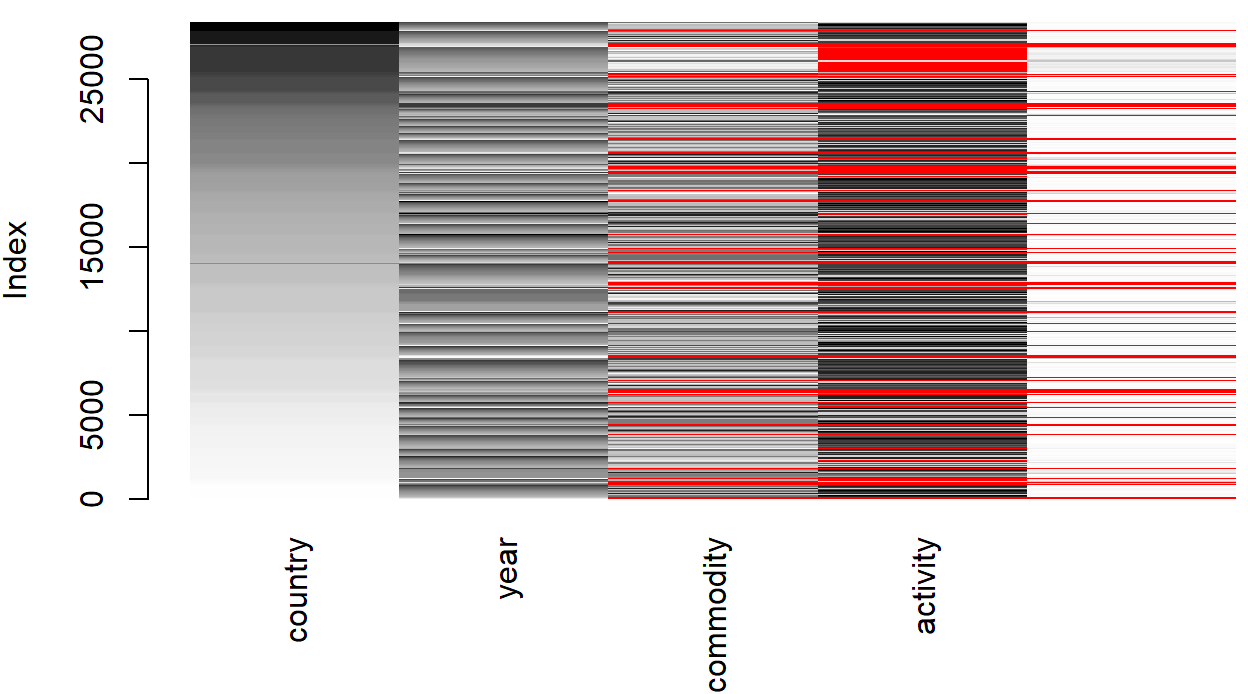


Figure S2. Missing Data Matrix Plot

This matrix plot provides a detailed visualization of missing data distribution across all observations (rows) and key variables (columns). Observed data are shown in grayscale, while missing values are highlighted in red. The figure reveals that missingness in *activity* and *commodity* is scattered throughout the dataset but also forms blocks in certain ranges of the index, likely corresponding to specific countries or years with partial data coverage. In contrast, *country* and *year* columns are complete for all records. This visualization underscores the unbalanced nature of the panel data structure, where data gaps are non-random and clustered, potentially contributing to volatility in temporal trend estimates for some commodity-activity combinations (Figure S2).


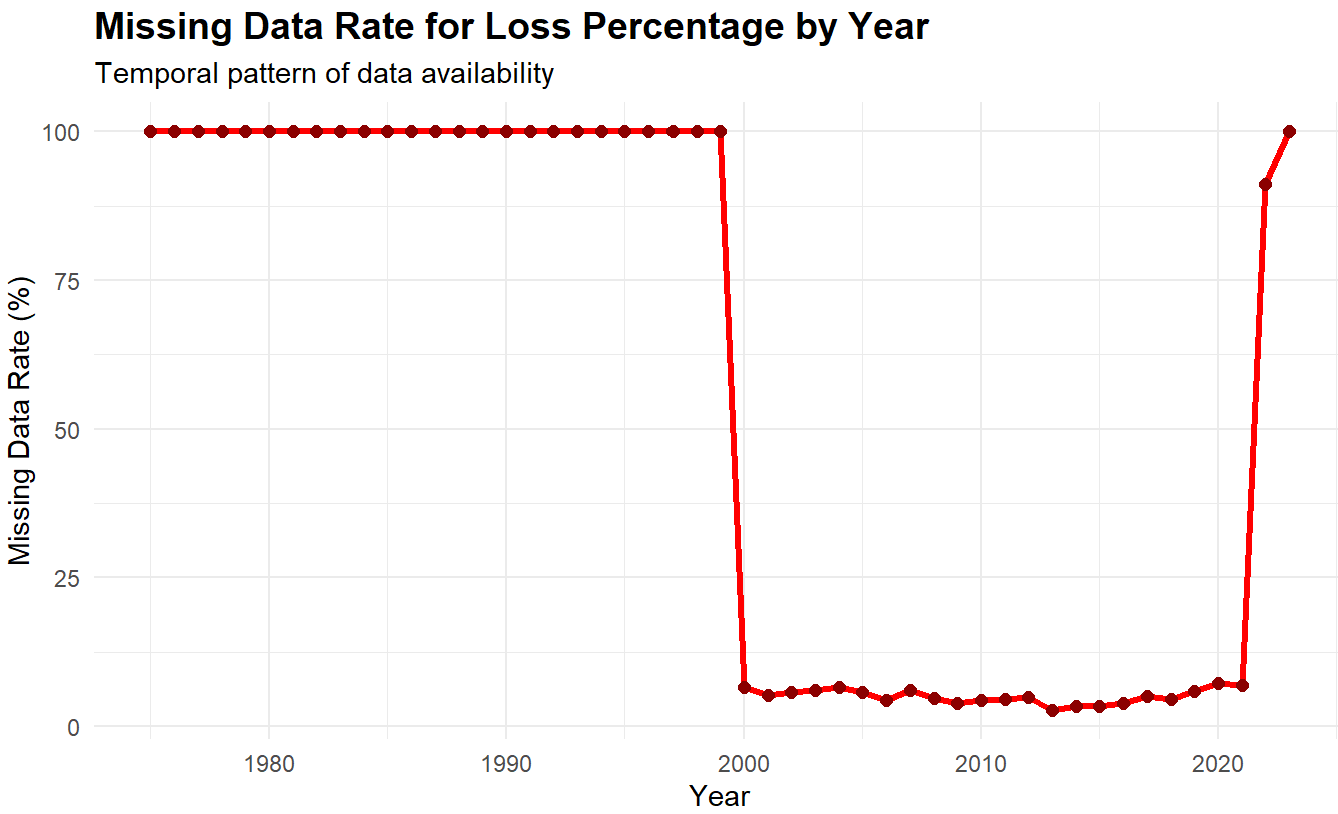


Figure S3. Temporal Pattern of Missing Data for Loss Percentage

This line graph shows the annual rate of missing data for the *loss percentage* variable from 1975 to 2023. The figure highlights two critical periods: (1) from 1975 to 1999, where loss percentage data are entirely missing (100% missing rate), and (2) from 2000 to 2021, where the missing rate drops sharply to ~5%–7%, indicating relatively good data availability during the core analysis period. However, after 2021, missing rates rise steeply again, reaching 91% in 2022 and 100% in 2023. This temporal pattern indicates that the robust findings reported in the study are primarily derived from the 2000–2022 period, where data completeness is highest, while caution is needed for interpreting trends at the dataset’s temporal boundaries (Figure S3).

**SUPPLEMENTARY CHAPTER 2:**

**EVALUATING TEMPORAL WEIGHTING VERSUS TIME COVARIATES IN COMMODITY AND ACTIVITY MODELS**

This sensitivity analysis was designed to evaluate whether applying a temporal weighting function would improve model fit in the multilevel mixed-effects models for food loss percentages (FLPs) across commodity and activity groups. The main analytical framework uses a linear covariate (Year – 2000) to control for temporal effects, but given the inherent imbalance in the panel structure—uneven year coverage, clustered observations in certain periods, and gaps in reporting—it was necessary to examine whether a weighted cross-sectional approach might better account for historical and recent data patterns.

$$w_{t}=\frac{e^{\lambda*\left[ t_{i}-\min\left( t \right) \right]}}{\sum_{s\in t} e^{\lambda*\left[ s_{t}-\min\left( t \right) \right]}}, \lambda>0$$

where

- **e:** Euler’s number;
- **ti:** the year of the corresponding observation;
- **min(t):** earliest year in the dataset (normalizes the timeline to zero),
- **λ:** a positive parameter (λ > 0) that controls the slope of the weighting function over time, fixed at 0.2;
- **st:** summation, which is performed over all available years (index s).

We tested a range of lambda values (0.00≤λ≤0.50) to assess whether progressively emphasizing recent years would yield more accurate estimates of FLPs across commodity groups and supply chain stages. The Akaike Information Criterion (AIC) was used to compare models incorporating the temporal weighting function against the original specification with (Year – 2000) as a covariate.

The results showed that for both commodity and activity models, the unweighted approach (λ=0.00\lambda = 0.00λ=0.00) consistently achieved the lowest AIC values. This finding indicates that incorporating year as a fixed covariate (Year – 2000) in a multilevel framework is more effective than applying temporal weights to FLP observations. Moreover, the unweighted specification allows for a more explicit interpretation of temporal effects and accommodates irregular year-to-year data gaps without introducing bias from arbitrary weighting. Thus, we retained the Year – 2000 covariate in the final models and did not apply any temporal weighting beyond the clustering phase for development typology.


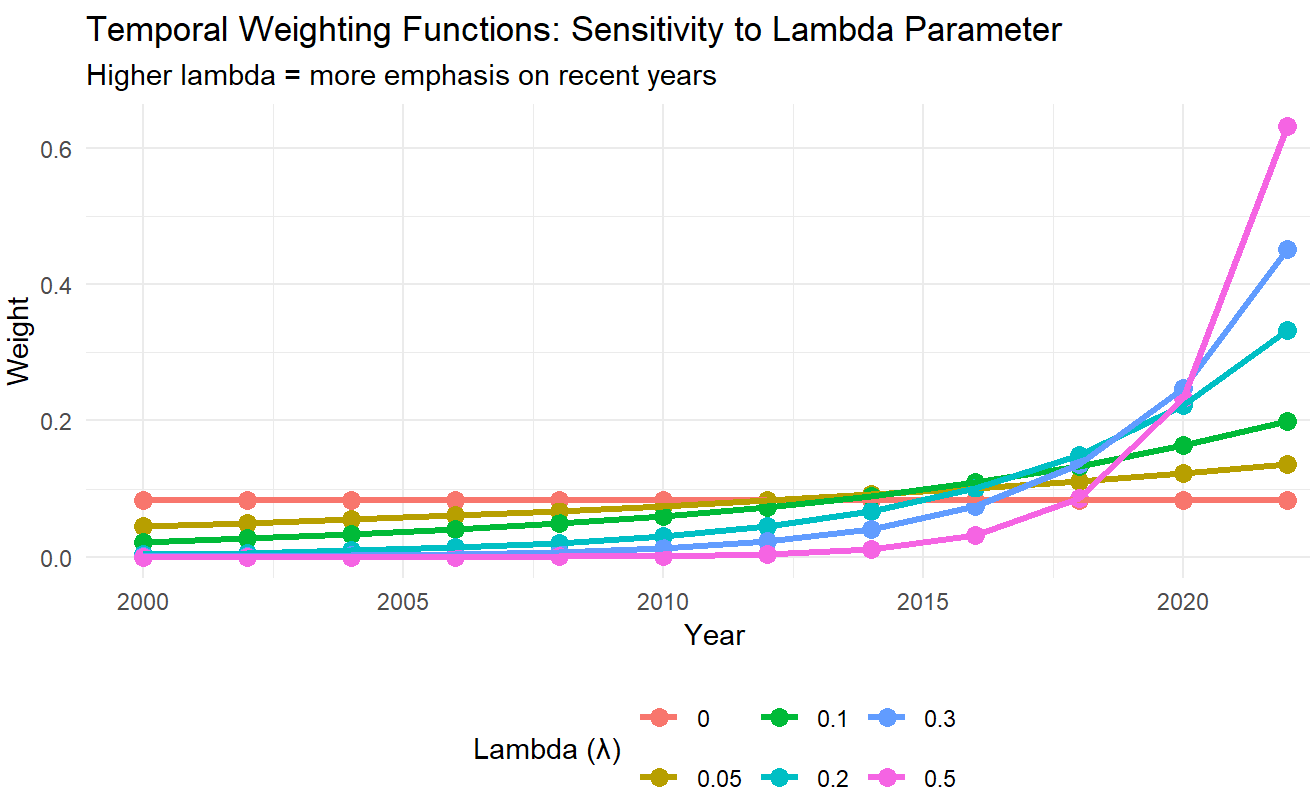


Figure S4. Sensitivity of the temporal weighting function to λ parameter

Figure S4 illustrates how increasing λ values in the temporal weighting function progressively amplify the influence of recent years.

Table S5 presents a detailed comparison of model performance metrics across temporal weighting functions applied with varying λ (lambda) values ranging from 0 to 0.5. For each λ, we report the mean Akaike Information Criterion (AIC), standard deviation of AIC (SD AIC), mean marginal R², and mean conditional R² values derived from Generalized Linear Mixed Models (GLMMs) fitted separately for four commodity groups and five activity groups. These metrics collectively assess whether increasing temporal emphasis improves model fit and generalizability in the presence of temporal heterogeneity within our dataset.

Table S5. Mean AIC and R² Values for Temporal Weighting Models (λ = 0–0.5)

|  | **Lambda (λ)** | **Mean AIC** | **SD AIC** | **Mean Marginal R²** | **Model Count** |
| --- | --- | --- | --- | --- | --- |
| **Commodity Groups** | 0.00 | 28.575 | 44.683 | 0.024 | 4 |
|  | 0.05 | 28.946 | 45.39 | 0.023 | 4 |
|  | 0.10 | 29.63 | 46.63 | 0.022 | 4 |
|  | 0.15 | 30.588 | 48.332 | 0.022 | 4 |
|  | 0.20 | 31.786 | 50.433 | 0.023 | 4 |
|  | 0.25 | 33.19 | 52.879 | 0.027 | 4 |
|  | 0.30 | 34.771 | 55.62 | 0.035 | 4 |
|  | 0.35 | 36.506 | 58.615 | 0.048 | 4 |
|  | 0.40 | 38.37 | 61.828 | 0.064 | 4 |
|  | 0.45 | 40.346 | 65.228 | 0.084 | 4 |
|  | 0.50 | 42.417 | 68.787 | 0.105 | 4 |
| **Activity Groups** | 0.00 | 25099 | 14732 | 0.029 | 5 |
|  | 0.05 | 25250 | 14752 | 0.033 | 5 |
|  | 0.10 | 25691 | 14912 | 0.037 | 5 |
|  | 0.15 | 26394 | 15214 | 0.041 | 5 |
|  | 0.20 | 27323 | 15660 | 0.045 | 5 |
|  | 0.25 | 28448 | 16246 | 0.050 | 5 |
|  | 0.30 | 29740 | 16964 | 0.056 | 5 |
|  | 0.35 | 31174 | 17800 | 0.061 | 5 |
|  | 0.40 | 32729 | 18743 | 0.067 | 5 |
|  | 0.45 | 34385 | 19776 | 0.073 | 5 |
|  | 0.50 | 36126 | 20888 | 0.081 | 5 |

The findings demonstrate a consistent pattern across both commodity and activity group models: the unweighted configuration (λ = 0) yielded the lowest mean AIC values—28,575 for commodity groups and 25,099 for activity groups—indicating the best penalized model fit. As λ increased, AIC values systematically worsened, suggesting that placing stronger emphasis on recent years degraded model performance. For instance, at λ = 0.2, mean AIC rose to 31,786 for commodity groups and 27,323 for activity groups, resulting in penalties of +3,211 and +2,224 AIC points respectively compared to the unweighted baseline. At the extreme, λ = 0.5 imposed even larger penalties (commodity: +13,842; activity: +11,027), underscoring the negative impact of aggressive temporal weighting (Table S5).

The findings reveal a clear and consistent trend: the unweighted models (λ = 0) achieved the lowest mean AIC values in both commodity groups (28,575) and activity groups (25,099), serving as the optimal configuration for penalized model fit. As λ increased, AIC values systematically worsened, indicating that stronger emphasis on recent years degraded model performance. At the extreme, λ = 0.5 imposed substantial penalties (commodity: +13,842; activity: +11,027 AIC points relative to λ = 0), underscoring the detrimental impact of aggressive temporal weighting. While mean marginal R² values exhibited slight increases with higher λ (commodity: 0.024 → 0.105; activity: 0.029 → 0.081), these trends likely reflect increased model flexibility and parameter tuning effects rather than substantive improvements in explanatory power. This pattern suggests that temporal weighting did not enhance the model’s ability to capture underlying variation in food loss percentages. The results indicate that applying temporal weights offers no empirical advantage for these models. The unweighted configuration (λ = 0) consistently outperformed alternative specifications in terms of model fit and parsimony, supporting its selection as the preferred approach for subsequent analyses (Table S5).

| 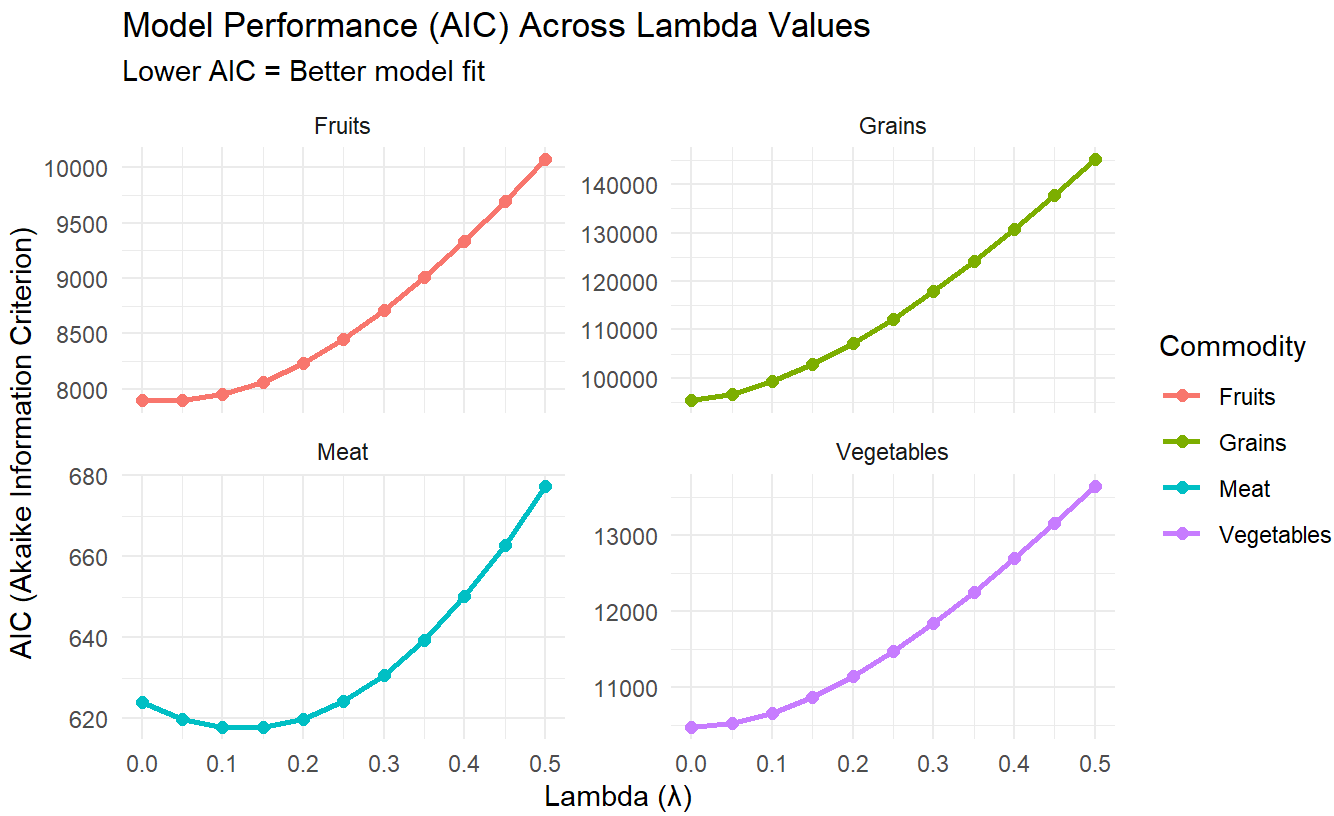 |
| --- |
| 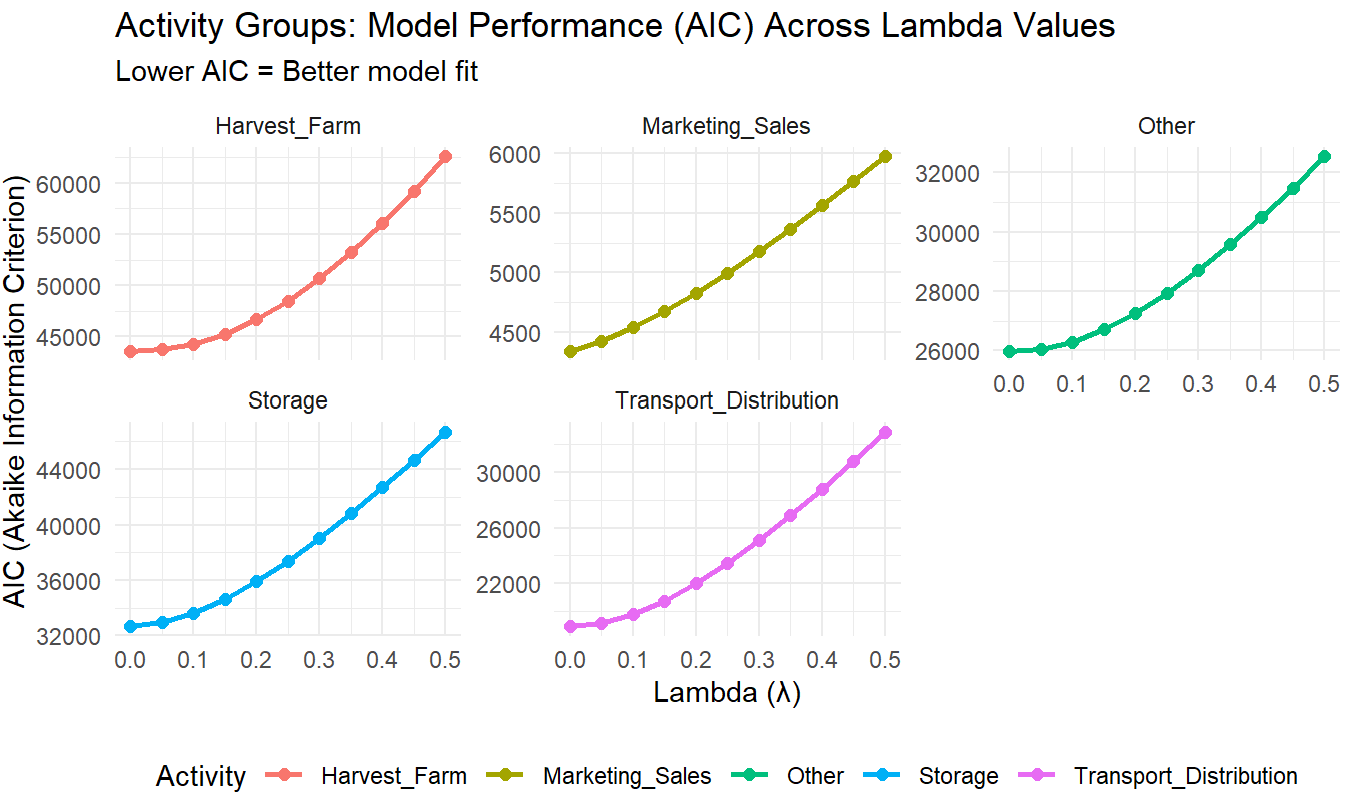 |

Figure S5. Model Performance (AIC) Across Lambda Values for Groups

As shown in Figure S5, Akaike Information Criterion (AIC) values systematically increase with higher λ values, indicating progressively poorer model fit as more emphasis is placed on recent years. For commodity groups, λ = 0 yielded the lowest mean AIC (28,575), while λ = 0.2 imposed a notable penalty of +3,211 AIC points. Similarly, activity group models exhibited their best performance at λ = 0 (mean AIC = 25,099), with λ = 0.2 again incurring a penalty of +2,224 AIC points (Figure S5).

In summary, contrary to initial theoretical considerations that temporal weighting might enhance sensitivity to recent trends, empirical evidence provides no support for applying it in these models. Both commodity and activity group analyses converge on the unweighted configuration (λ = 0) as the most robust and parsimonious specification. Therefore, all reported results in the main text are based on unweighted GLMMs, which account for temporal heterogeneity via explicit linear time covariates (Year – 2000) and random effects without introducing unnecessary complexity or compromising model stability.
